# Pitfalls of Establishing DNA Barcoding Systems in Protists: The Cryptophyceae as a Test Case

**DOI:** 10.1371/journal.pone.0043652

**Published:** 2012-08-24

**Authors:** Kerstin Hoef-Emden

**Affiliations:** Botanical Institute, Cologne Biocenter, University of Cologne, Cologne, Germany; Université Paris Sud, France

## Abstract

A DNA barcode is a preferrably short and highly variable region of DNA supposed to facilitate a rapid identification of species. In many protistan lineages, a lack of species-specific morphological characters hampers an identification of species by light or electron microscopy, and difficulties to perform mating experiments in laboratory cultures also do not allow for an identification of biological species. Thus, testing candidate barcode markers as well as establishment of accurately working species identification systems are more challenging than in multicellular organisms. In cryptic species complexes the performance of a potential barcode marker can not be monitored using morphological characters as a feedback, but an inappropriate choice of DNA region may result in artifactual species trees for several reasons. Therefore *a priori* knowledge of the systematics of a group is required. In addition to identification of known species, methods for an automatic delimitation of species with DNA barcodes have been proposed. The Cryptophyceae provide a mixture of systematically well characterized as well as badly characterized groups and are used in this study to test the suitability of some of the methods for protists. As species identification method the performance of blast in searches against badly to well-sampled reference databases has been tested with COI-5P and 5′-partial LSU rDNA (domains A to D of the nuclear LSU rRNA gene). In addition the performance of two different methods for automatic species delimitation, fixed thresholds of genetic divergence and the general mixed Yule-coalescent model (GMYC), have been examined. The study demonstrates some pitfalls of barcoding methods that have to be taken care of. Also a best-practice approach towards establishing a DNA barcode system in protists is proposed.

## Introduction

DNA barcodes are short and highly variable DNA regions that can be used to identify species [1]. The COI-5P region, encompassing 500 to 800 nucleotides of the 5′ terminus from the mitochondrial gene *cox*1, became a standard barcode in zoology [2]. *Cox*1 codes for subunit I of cytochrome c oxidase (complex IV of the respiratory chain). But since discriminative power of COI-5P proved to be insufficient in some eukaryotic groups, also other organellar protein-coding genes, such as e.g. plastid-encoded *mat*K and subunits of *rpo* (in embryophyte plants), or regions of the eukaryotic ribosomal operon, e.g. 5′-partial LSU rDNA, ITS2 or the V4 region of the SSU rRNA gene (e.g. in diatoms, foraminifera, green algae or fungi), have been proposed as alternatives [3]–[7].

To work accurately, DNA-based identification methods require a dense taxon sampling, but also a consistent systematics [8]. Funk and Omland summarized problems in DNA barcoding of animals that will likely cause failures in species identification due to poly- or paraphylies in species that can be roughly assigned to three categories [9]: (a) an artificial systematics combined with an appropriate barcode marker (termed “imperfect taxonomy” by Funk and Omland), (b) a consistent systematics, but an inappropriate molecular marker and (c) naturally non-monophyletic taxa. Causes for category (a) may be the use of unspecific or misleading morphological characters for species delimitation. The inferred phylogeny may be correct, but species names are not congruent with biological species. In category (b) genes have been either subject to introgression, to incomplete lineage sorting after duplication events or paralogous instead of orthologous gene copies have been used as barcode markers. In these cases systematics may be consistent, but the wrong marker has been chosen for barcoding. Its gene phylogeny does not reflect the species tree. Funk and Omland did not mention lateral/horizontal gene transfer that also could result in a gene tree deviant from the species tree, probably because these events have been reported from eukaryotes only rarely, e.g. for mitochondrial genes in embryophyte plant species [10]. Problems of categories (a) or (b) can be solved by a taxonomic revision or by choosing an alternative barcode marker, respectively. Difficulties arise, if biological species are naturally non-monophyletic (c). Natural non-monophyly may be caused by interspecific hybridization events or by reproductive isolation of a subpopulation from a species. In the latter case, the parent species will be paraphyletic with the monophyletic offspring species being nested inside. Apart from the problems listed by Funk and Omland, molecular traits inherent in a chosen marker may also result in artifactual tree topologies or erroneous species identifications, e.g. unequal evolutionary rates across taxa, sites and/or time, heterogenous base compositions or differing codon usage [11]–[15].

Once a reference database has been set up, species identification can be performed by using a query sequence to search a reference database [16]. Restricting a search to a group of organisms reduces the risk of species misidentifications, because substitution saturation of a marker is minimized. Whereas morphological characters seem to reflect to some extent biological species limits in animals, phenotypic characters proved to be misleading in many protistan lineages [17]–[22]. As a consequence, researchers who want to establish DNA barcoding systems for protists face several challenges. In many lineages, a chaotic classification with poly- or paraphyletic species prevails, but for an accurate identification of species a consistent systematics is required and in addition *a priori* knowledge of species phylogeny often provides the only means for a plausibility control to identify problems such as introgression or incomplete lineage sorting in a candidate barcode marker. Problems arise also in searches against reference databases. Similar cellular organizations of protists – colorless flagellates, amoebae, green kokkoids – are spread across supergroups/kingdoms in the eukaryotic tree of life (Plantae/Archaeplastida, Unikonta, Excavata, cryptophytes/haptophytes and the SAR group with alveolates, stramenopiles, Rhizaria) [23]–[25]. Thus, constraining a search to the correct group requires expertise and training in light microscopical methods. On the other hand, nanoplanktonic protists may be too small to be identified by light microscopy and often only environmental DNA sequences have been sampled to assess microcosmic diversity in a habitat, e.g. [26]–[30]. Both does not allow for restricting searches.

Phylogenetic methods unveiled cases of inconsistent systematics at different classification levels in protistan lineages, resulting in taxonomical revisions that combined classical methods such as light and electron microscopy with molecular phylogenetic analyses and, if possible, also with mating experiments [31]–[35]. For an unambiguous identification of taxa, molecular synapomorphies, molecular signatures or accession numbers to sequences have been included into taxonomic descriptions [33] [36]–[37]. For taxa that can not be maintained in laboratory culture, isolation of single cells, subsequent morphological observation, single-cell PCR and subsequent sequencing or *in situ* FISH hybridization methods have been proposed to facilitate integrative taxonomy [38]–[39].

Some protists, e.g. the Euglenida, seem to reproduce only asexually. In others, sexual reproduction is known or suspected, but they can not be grown in laboratory culture or their inductors of sexual reproduction are not known. Only in few protistan lineages mating experiments have been possible. Annette Coleman performed crossing experiments in volvocalean algae and compared the results with sequences and secondary structures of the internal transcribed spacer 2 (ITS2) of the eukaryotic ribosomal operon [40]. In most eukaryotes ITS2 folded up to four major helices with the proximal part of helix 2 and helix 3 being the most conserved regions, important for the splicing process of the pre-rRNA transcript [41]–[42]. If a compensatory base pair change (CBC) occurred in one of the most conserved parts of the ITS2 secondary structure, two cultured algal strains proved to be separate biological species that did not produce fertile offspring [40]. A similar correlation has been found in other groups across the eukaryotic tree (e.g. in diatoms, desmids, brown algae, embryophyte plants, animals, fungi; summarized in [43]). CBCs in ITS2 seem to be a safe predictor for sexual incompatibility, albeit by chance. The nuclear ribosomal operon is not directly linked to mating. Due to unequal evolutionary rates across lineages, the CBC clade concept probably does not work in both directions (i.e. a CBC clade may encompass several biological species). Therefore CBCs in the ITS2 can be considered only an approximation to biological species limits [44]–[45]. The use of ITS2 for a delimitation of species has been spreading in protists since [20] [32] [46], and in fungi, species identification by ITS2 even precedes the use of COI-5P as a DNA barcode [44]. However, ITS regions may be subject to intragenomic variation, resulting in artifactual increases of species counts, which cautions against the use of ITS2 as a barcode marker [47].

Considering the vast diversity of protists across the eukaryotic tree of life, systematics research at low classification levels in protists is still in its infancy [48]–[49]. For some Cryptophyceae larger taxonomic revisions based on integrative taxonomy have been published previously [20] [33] [50]. Cryptomonads are biflagellate and mostly photoautotrophic unicells that occur ubiquitously in freshwater, brackish and marine environments [51]. Their complex plastids originate from a secondary endosymbiosis with a red alga [52]. The former nucleus of the engulfed alga, termed the nucleomorph, has been retained between the two outer and the two inner plastid membranes [53]. The plastid-bearing cryptomonads (cryptophytes), thus, contain four genomes in a cell, two of eukaryotic and two of prokaryotic origin (nucleus, nucleomorph, plastid and mitochondrial genome), each with its own ribosomal RNA genes.

Apart from the phagotrophic and aplastidic genus *Goniomonas* Stein and a lineage with an unknown mode of nutrition inferred from environmental sequencing, five clades and two isolated lineages are known (the genus *Cryptomonas* Ehrenberg: freshwater only; the *Rhodomonas* clade with the genera *Rhodomonas* Karsten, *Rhinomonas* Hill et Wetherbee and *Storeatula* Hill: predominantly marine; the *Chroomonas* clade with *Chroomonas* Hansgirg, *Komma* Hill and *Hemiselmis* Parke: marine or freshwater; the *Teleaulax* clade with *Geminigera* Hill, *Plagioselmis* Butcher, *Teleaulax* Hill: predominantly marine; the *Guillardia* clade with *Guillardia* Hill et Wetherbee and *Hanusia* Deane, Hill, Brett et McFadden; the marine monospecific genera *Falcomonas* Hill and *Proteomonas* Hill et Wetherbee) [54]. Most of the clades were mixtures of poly- or paraphyletic genera, likely due to unidentified heteromorphic life cycles indicative also for sexual reproduction [33] [55].

Cryptophytes as such can be easily identified by their asymmetric cell shape and subapical flagellar insertion site causing a wavy rotation along the longitudinal axis during swimming. Around 200 species have been described, but proved to be based on unreliable or unspecific morphological characters in the genera *Cryptomonas* and *Hemiselmis* [20] [50]. Only the latter genera have been revised to date [20] [33] [50]. The two revisions of the genus *Cryptomonas* have been based on comprehensive sampling of 122 clonal strains, ordered from diverse culture collections around the world and newly isolated [20] [33]. For a representative subset of these strains completely congruent data sets comprising 5′-partial 28S rDNA and ITS2 of the nuclear ribosomal operon and the 18S rRNA gene of the nucleomorph ribosomal operon have been published. Since nuclear and nucleomorph ribosomal operons are not linked, potential artifacts, e.g. caused by base composition biases or unequal evolutionary rates, could be ruled out by examining single-gene phylogenies of nuclear and nucleomorph genes for conflicting branching patterns. Whereas in the first revision, species level has been assigned to terminal clades (except for *Cryptomonas borealis* CBCs proved to be absent in the ITS2 sequences of the species), the second revision was entirely based on the CBC concept [20]. For the unrevised *Chroomonas* clade a large congruent data set of nuclear 5′-partial LSU and SSU rDNA and of nucleomorph SSU rDNA was also available [56].

The previously published highly resolved and comparably densely sampled phylogenies of the genus *Cryptomonas* and of the *Chroomonas* clade provided the means to monitor the performance of the tested barcode markers as an alternative to misleading morphology. This study addresses species identification and potential species delimitation methods in cryptophytes. Some weaknesses in barcoding methods are demonstrated that should be taken care of when establishing a barcode system in protists.

Blast searches against a reference database belong to the species identification methods. The INSDC databases represented the most comprehensive archive of sequenced taxa. Data of the Barcode of Life database BOLD*Systems* have been imported, whereas BOLD*Systems* included only a subset of sequences from INSDC, namely animal, fungal and plant barcodes [16] [57]. To date people working on protists are, thus, forced to use NCBI’s blast tools for an identification of taxa at different classification levels. The performance of candidate DNA barcodes using cross-eukaryote searches have been tested under different sampling scenarios in this study. As queries, a cryptophyte COI-5P sequence and 5′-partial nuclear LSU rDNA sequences of differently well sampled clades in the cryptophytes have been used. Of the 21 novel sequences in this study (eleven 5′-partial nuclear LSU rDNA sequences in the *Chroomonas* clade, eight 5′-partial nuclear LSU rDNA sequences in the genus *Cryptomonas*, one 5′-partial nuclear LSU rDNA sequence of a *Rhodomonas* strain and a new COI-5P sequence of a *Cryptomonas* species), only sequences representing new lineages at different classification levels have been used in the blast searches.

Frequency distributions of genetic distances have been used to identify a barcode gap assumed to separate intra- from interspecific frequency distributions [2]. Thus, this method has been suggested for species delimitation. Based on the barcode gap, thresholds of genetic divergence in percent have been proposed to identify new species. This practice has been shown not to work in all cases and to have been biased by wrong computation of frequency distributions [8] [58]. Several alternative methods for an automatic delimitation of species, thus, have been invented. Pons et al. proposed the general mixed Yule-coalescent (GMYC) model for a large scale delimitation of species [59]. The authors stressed that, different from other introduced methods for automatic species delimitation, no *a priori* knowledge of biogeographic distribution was required for GMYC. Since protists are usually not biogeographically distributed [60]–[61], this method could be suited for species delimitation in protistan lineages. GMYC requires an ultrametric tree to generate a plot showing the increase of lineages during the course of time (lineage-through-time plot, LTT) and to identify a switch in branching rates indicating a change from speciation events to coalescence (intraspecific variation at population level) [59] [62]. Pons et al. termed the time intervals from one to the next speciation event “waiting times”. The threshold between speciation and coalescence is found in a maximum likelihood computation by optimizing the sum of log-likelihoods of waiting times across the tree. To assess whether a differentiation between speciation and coalescence during the course of time is more probable than the notion of coalescence events only (i.e. all terminal nodes represent members of one population) a likelihood ratio test can be performed with coalescence-only as a null hypothesis [59]. Since most sequences do not evolve following a strict molecular clock, Monaghan et al. suggested to use a relaxed clock for tree inference instead and also invented a multiple threshold algorithm for GMYC that allowed for different switching times from speciation to coalescence adjusted to different evolutionary lineages across the tree [63].

In this study, 5′-partial LSU rDNA is used to assess the before-mentioned two different methods of automatic species delimitation. Phylogenetic trees with this DNA region were congruent with the phylogenies derived from nucleomorph SSU rDNA, thus, tree artifacts as mentioned above are unlikely. Two different 5′-partial LSU rDNA data sets (sequences of the *Chroomonas* clade and an alignment of *Cryptomonas* sequences) have been used to examine how evolutionary models and changes in taxon sampling shape a frequency distribution of genetic distances and the position and shape of potential barcode gaps. Also species delimitation by the GMYC model, both with the multiple threshold and the single threshold models have been tested with the better sampled *Cryptomonas* data set. Usually, the ultrametric trees for GMYC have been calibrated. Since no fossil record was available for cryptophytes, an uncalibrated tree has been used instead.

## Results

### Species Identification

#### Performance of blast algorithms in an extremely badly sampled group using COI-5P as a query

The National Center of Biotechnology Information (NCBI) offers three different settings for nucleotide versus nucleotide queries with different scoring and word size schemes (Table 1) [64]–[65]. Per default, the search strategy is set to “megablast”, an algorithm supposed to be used for highly similar sequences, i.e. for intraspecific comparisons with identities equal or higher than 95%. At time of the blast experiments, only four cryptophyte COI-5P or complete *cox*1 sequences have been available in the INSDC databases, only one of them, *Cryptomonas ovata* strain NIES-274 (acc. no. AB009419), belonged to the same genus as the query sequence (*Cryptomonas curvata* strain CCAC 0080; Table 2). Thus, the COI-5P sequences of the class Cryptophyceae represented an example for a bad sampling driven to the extreme.

**Table 1:** Scoring schemes of different nucleotide versus nucleotide search algorithms from the NCBI blast pages.

Search algorithm	Word size	Matches	Mismatches	Gap costs
Megablast	28	1	−2	Existence: 0, Extension: −2.5
discontiguous megablast*	11	2	−3	Existence: −5, Extension: −2
Blastn	11	2	−3	Existence: −5, Extension: −2

Megablast is supposed to perform best in comparisons of closely related sequences with identities beyond 95%, whereas discontiguous megablast and the slower blastn algorithm should be better suited for cross-species comparisons. All blast types used a default expectancy threshold of 10.

1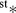, settings of discontiguous megablast differed from previously reported settings [64].

**Table 2:** Performance of blast searches in a badly sampled group using the COI-5P sequence of *Cryptomonas curvata* strain CCAC 0080 as a query (570 nt): rankings and statistics of the discontiguous megablast search.

Rank	Description	Score bits	Expectancy	Identities	Gaps	Coverage
1	*Cryptomonas ovata* mitochondrial COXI gene (AB009419.1)	580 (642)	4E–162	470/569 (83%)	0/569 (0%)	99%
2	*Macrocystis pyrifera* haplotype H5 (HM153261.1)	509 (564)	7E–141	455/570 (80%)	0/570 (0%)	100%
24	*Guillardia theta* strain CCMP 2712 (GQ896379.1)	489 (542)	6E–135	452/570 (79%)	2/570 (0%)	99%
>500	*Hemiselmis andersenii* strain CCMP 644 mitochondrion, completegenome (EU651892.1)	443 (490)	8E–121	434/560 (78%)	0/560 (0%)	98%
>1000	*Rhodomonas salina* mitochondrial DNA, completegenome (AF288090.1)					
	*R. salina* 1	113 (124)	8E–102*	76/85 (89%)	0/85 (0%)	94%
	*R. salina* 2	379 (420)	–	359/457 (79%)	1/457 (0%)	–

Except for *C. ovata* and *G. theta*, no other cryptophytes among first 100 hits; all others: stramenopile sequences, predominantly brown algae. Discontiguous megablast and blastn resulted in the same ranking among first hits. No changes were observed with blastn word size reduced to 7. The *R. salina cox*1 sequence was interrupted by a group 2 intron in the COI-5P region, thus yielded two alignments. Its low ranking did not allow for viewing of pairwise alignments. Query coverage and expectancy values according to list view, but score bits, identities, and gaps statistics have been taken from a search constrained to cryptomonads (expectancy values change under these circumstances).

When using the megablast algorithm, stramenopile sequences, but no cryptophyte sequences appeared among the first 100 hits (Table 2). Top hit was an oomycete, *Aphanomyces laevis*, second and third hits were brown algae of the order Fucales. In all cases the megablast algorithm inserted many gaps (up to 6% of the covered query) in the pairwise alignments, usually severely violating the triplet structure of the genes. The maximum identity of 80% of the three top positions was below the threshold of 95% considered the minimum for megablast to operate well. Constraining the megablast search to “cryptomonads (taxid:3027)” yielded no hits at all.

Switching to the discontiguous megablast algorithm resulted in a shuffling of positions. First hit was the only available *Cryptomonas* COI-5P sequence (*C. ovata* strain NIES-274). The next cryptophyte sequence, however, was ranked only in position 24 (*Guillardia theta* strain CCMP2712; Table 2). All other first 100 hits were stramenopiles with the giant kelp *Macrocystis pyrifera* in positions 2 to 10. The third cryptophyte sequence, *Hemiselmis andersenii* CCMP644 showed up somewhere between positions 500 and 1000, and the fourth cryptophyte sequence *Rhodomonas salina* in a position between 1000 and 5000 (positions have been inferred by increasing step-wise the number of hits to be shown, followed by a search for the genus name in the listing).

Only when using the blastx algorithm (translation of the query sequence to amino acids and comparing it to a protein database), all four cryptophyte COI-5P sequences appeared among the first 100 hits, but not in consecutive order in top four positions. Whereas the sequences of *C. ovata* strain NIES-274 and *G. theta* strain CCMP2712 were placed in positions 1 and 2, *H. andersenii* strain CCMP644 and *R. salina* appeared only in positions 8 and 31, respectively (blastx results not shown). Different from the nucleotide versus nucleotide searches, viridiplant taxa, embryophyte plants and Chlorophyta, prevailed among first 100 hits.

The megablast and discontiguous megablast results indicated that the sequences in GenBank may have reached substitution saturation. Thus, a data set with 12 COI-5P sequences was assembled (the new COI-5P sequence of *C. curvata*, the four cryptophyte sequences and the non-cryptophyte sequences flanking each cryptophyte entry from beyond and below) to examine genetic distances under different evolutionary models and to perform a test for substitution saturation according to Xia and Lemey [66] (Tables 3 and 4). Since the cryptophyte *Rhodomonas salina* was embedded between spider sequences, the taxon sampling finally crossed three supergroups/kingdoms, the Hacrobia (cryptophytes), the remains of the “chromalveolates” *alias* SAR group (stramenopiles) and the Opisthokonta (spiders).

**Table 3:** K2P and K2P and GTR+I+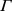 distances computed by maximum likelihood in *Cryptomonas curvata* CCAC 0080 COI-5P versus cryptophyte and non-cryptophyte sequences.

Rank	Description	K2P	K2P (ML)	GTR+I+Γ
1	*Cryptomonas ovata* (Hacrobia, Cryptophyceae; acc. no. AB009419)	0.200	0.209	0.293
2	*Macrocystis pyrifera* (stramenopiles, Phaeophyceae; acc. no. HM153261)	0.235	0.252	0.385
23	*Aphanomyces laevis* (stramenopiles, Oomycetes; acc. no. HQ708195)	0.250	0.270	0.437
24	*Guillardia theta* (Hacrobia, Cryptophyceae; acc. no. GQ896379)	0.250	0.267	0.427
25	*Saccharina coriacea* (stramenopiles, Phaeophyceae; acc. no. AP011499)	0.255	0.272	0.427
>500–1	*Pterygophora californica* (stramenopiles, Phaeophyceae; acc. no. FJ409188)	0.272	0.292	0.479
>500	*Hemiselmis andersenii* (Hacrobia, Cryptophyceae; acc. no. EU651892)	0.275	0.296	0.497
>500+1	*Alaria praelonga* (stramenopiles, Phaeophyceae; acc. no. EF218902)	0.272	0.292	0.465
>1000–1	*Nephila pilipes* (Metazoa, Chelicerata; acc. no. AY052597)	0.318	0.350	0.660
>1000	*Rhodomonas salina* (Hacrobia, Cryptophyceae; acc. no. AF288090)	0.255	0.270	0.428
>1000+1	*Agelenopsis aleenae* (Metazoa, Chelicerata; acc. no. AY770786)	0.313	0.345	0.678

The non-cryptophyte sequences have been found one position below or beyond a cryptophyte sequence in the discontiguous megablast hit list and have been aligned with the cryptophytes to infer genetic distances (11 taxa from discontiguous megablast search and the query sequence; 570 positions). In the spider sequences two gaps had to be inserted, corresponding to two codons.

**Table 4:** Results of the tests for substitution saturation.

Data set	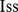	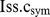	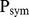	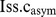	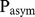
COI-5P, 1 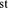 and 2 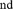 codon positions only	0.252	0.700	0.000	0.522	0.000
COI-5P, 3 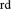 codon positions only	0.690	0.675	0.501	0.508	0.000
5′-partial LSU rDNA, *Chroomonas* clade	0.244	0.741	0.000	0.436	0.000
5′-partial LSU rDNA, genus *Cryptomonas*	0.253	0.749	0.000	0.436	0.000
5′-partial LSU rDNA,combined data	0.268	0.736	0.000	0.421	0.000

Proportions of invariant sites have been taken from maximum likelihood estimators, since DAMBE crashed during their computation: COI-5P, 1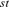 and 2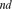 codon positions  = 0.283; COI-5P, 3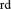 codon positions  = 0.000; 5′-partial LSU rDNA, *Chroomonas* clade  = 0.652; 5′-partial nuclear LSU rDNA, genus *Cryptomonas*  = 0.627; 5′-partial LSU rDNA, combined data sets, including *Rhodomonas* sp. strain CCAP 978/13; *Rhodomonas* sp. strain M1480 (acc. no. AM396399) and *Storeatula* sp. strain CCMP1868 (acc. no. FJ973366)  = 0.594. 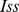, index of substitution saturation as inferred from the mean value of sitewise entropies (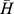) divided by global entropy of nucleotides (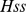); 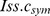, saturation threshold of 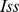 for symmetrical trees; 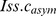, saturation threshold for asymmetrical trees. If 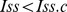 and difference between 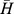 and 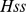 is significant (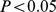), saturation probably is low in the data set. If 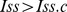 and the difference between 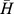 and 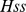 is significant, the data set is useless for phylogenetic analyses. If 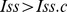 and the difference between 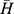 and 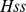 is not significant, the quality of the data set for phylogenetic analyses is poor. Resampling of OTUs is required for data sets larger than 32 taxa, because no critical 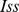 values for larger data sets were available from simulations [67] [66]. For the 5′-partial LSU rDNA data sets, the values for resampling of 32 taxa are shown.

K2P inferred by maximum likelihood resulted in slightly higher values than K2P distances estimated by the maximum likelihood parameters in the COI-5P data set (Table 3). The steepest increase in distances was found in the GTR+I+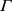 model. Under all three settings, *C. ovata* proved to be the closest relative to *C. curvata* with distances of 0.200 (K2P), 0.209 (K2P estimated by ML) and 0.293 (GTR+I+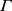). Under none of the evolutionary models, however, ranking order approximated correct relationships. The giant kelp *Macrocystis pyrifera* always seemed to be the next-closest relative to *C. curvata* and *Hemiselmis andersenii* was always in 9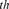 position with 5 non-cryptophyte sequences seemingly closer related to *C. curvata*. Only in the genetic distances to *R. salina* and its neighboring discontiguous megablast hits, two spider sequences (*Nephila pilipes* and *Agelenopsis aleenae*), differences indicated that the simple blast scoring scheme may be more limited than an evolutionary model. If the position of *R. salina* would have been inferred by GTR+I+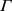 modeled distances, it would have been ranked close to *G. theta*. Neighbor-joining tree inference, however, proved to be slightly more robust than a simple ranking according to distances. Under K2P as well as under GTR+I+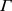, the cryptophyte sequences were monophyletic with high bootstrap support (both: 94%; 1000 replicates), but the oomycete *Aphanomyces laevis* grouped with high support with the two spider sequences (K2P: 91.1%; GTR+I+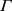: 91.4%) (trees not shown).

For all data sets the proportions of invariable sites have been determined prior to the tests for substitution saturation and gapped sites have been excluded. In COI-5P, the tests have been performed separately for a data set consisting only of 1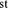 and 2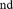 codon positions and for a data set comprising 3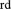 codon positions (Table 4). Whereas the data set with 1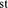 and 2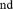 codon positions passed the test (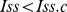 and difference between 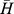 and 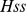 significant; see Table 4), a high substitution saturation could be confirmed for 3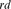 codon positions. Assuming a symmetrical tree topology, the differences between 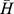 and 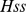 proved to be not significant, meanwhile the ratio of *Iss* versus 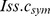 approached 1 or reversed (Table 4). This combination indicated a considerable saturation. For asymmetrical trees the situation was worse. 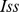 became larger than 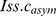 with a significant difference between 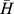 and 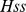, meaning that the most important positions for species delimitation were useless for phylogenetic analyses. Since these 13 taxa crossed three eukaryote supergroups/kingdoms, a phylogenetic tree likely would have been considerably asymmetric.

#### The impact of gap costs on the performance of blast searches in a protein-coding gene

**Figure 1:**
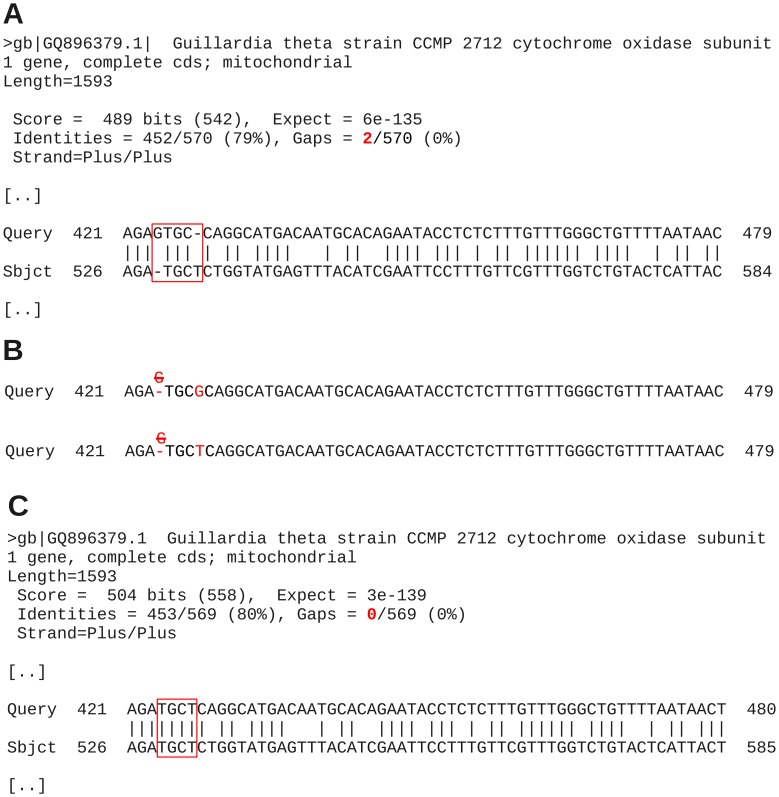
Examples for pairwise alignments of the COI-5P query sequence of *Cryptomonas curvata* strain CCAC 0080 with the *Guillardia theta* COI-5P region using the discontiguous megablast algorithm. **A** – Discontiguous megablast inserted two gaps into the alignment at high gap costs. **B** – Manual modifications of the query sequence to test the impact of gaps on ranking. Top row: Shifting the G (red) by three positions downstream resulted in disappearance of both gaps with one mismatch. Bottom row: Deletion of a G and insertion of a T three nucleotides downstream resulted in a perfect match in this short DNA stretch. **C** – Result of a discontiguous megablast search using the modified query sequence with an exact matching four-nucleotide string.

In the pairwise alignment of *C. curvata* with *G. theta* CCMP2712 two gaps have been inserted by discontiguous megablast to facilitate matching of three nucleotides – TGC – between query and *G. theta* sequence (Fig. 1A; Table 2). One gap has been inserted in the *G. theta* sequence upstream of these three nucleotides, the other gap in the query sequence downstream, thus disturbing the open reading frames of both genes (Fig. 1A). To examine the impact of the two gaps on the ranking position of *G. theta*, two test searches under elimination of the gaps have been performed. Since the published *G. theta* sequence itself could not be modified, the query sequence was altered manually instead (Fig. 1B; modified positions labeled in red). The G placed in position 424 of the query sequence was deleted and the gap position that has been inserted between positions 427 and 428 was filled with a G or a T (Fig. 1B). In the first case, the G would result in a mismatch (Fig. 1B, top row) and in the latter case, query and *G. theta* sequences would exactly match in the modified part (Fig. 1B, bottom row). As a consequence of these slight modifications in the *C. curvata* sequence, the ranking of the *G. theta* sequence improved from rank 24 (489 score bits) up to positions 7 (mismatch with a G; 499 score bits; results not shown) or 5 (match with a T; 504 score bits; Fig. 1C and Table 5). The slightly different query sequence expectedly resulted in changes of scoring and expectancy values, but sequence entries of positions 1 to 4, query coverage and maximum identities remained the same (Table 5). In the case of an exact matching region between query sequence positions 424 and 427, the *G. theta* sequence obtained the same overall scoring and expectancy values as the *Macrocystis pyrifera* clones. It differed from the latter only in maximum identity by 1%. Thus, the insertion of only two single-position gaps with a total penalty of −10 into an alignment had an extreme impact on ranking position in a conserved protein-coding gene usually devoid of gaps in closely related groups such as COI-5P. A search with a lower gap cost of 2 resulted in position 25 with even more gaps inserted into the pairwise alignment of *C. curvata* and *G. theta*. A single sequencing error, thus, may have fatal effects.

**Table 5:** The impact of gap costs in a saturated DNA barcode marker: discontiguous megablast search using a manually modified *Cryptomonas curvata* CCAC 0080 COI-5P sequence as a query.

Rank	Description	Score bits	Expectancy	Identities	Gaps	Coverage
1	*Cryptomonas ovata* mitochondrial COXI gene (acc. no. AB009419.1)	576 (638)	5E–161	469/569 (82%)	0/569 (0%)	99%
2	*Macrocystis pyrifera* haplotype H5 (acc. no. HM153261.1)	504 (558)	3E–139	456/571 (80%)	0/571 (0%)	100%
3	*Macrocystis pyrifera* haplotype H4 (acc. no. HM153260.1)	504 (558)	3E–139	456/571 (80%)	0/571 (0%)	100%
4	*Macrocystis pyrifera* haplotype H3 (acc. no. HM1532590.1)	504 (558)	3E–139	456/571 (80%)	0/571 (0%)	100%
5	*Guillardia theta* strain CCMP2712 (acc. no. GQ896379.1)	504 (558)	3E–139	453/569 (80%)	0/569 (0%)	99%

Shown: Modification with an exact match between positions 424 and 427 of the 570 nt query sequence (see text and Fig. 5).

Query coverage between the *C. curvata* COI-5P sequence and the worst-ranked cryptophyte sequence, *Rhodomonas salina*, was 4 to 5% lower than between *C. curvata* and the other cryptophytes. The output also indicated a split between positions 110 and 111 of the query sequence into two separate alignments (Table 2). Both may add to the low ranking. According to annotation a group II intron separated the cox1 gene in the COI-5P region between positions 34837 and 38929 of the *R. salina* mitochondrial genome (acc. no. AF288090).

#### Performance of blast algorithms in 5′-partial nuclear LSU Rdna

Three scenarios have been set up to test the performance of blast with 5′-partial nuclear LSU rDNA as a candidate barcode marker: (a) using a query sequence from one of the badly sampled large clades within the cryptophytes; (b) using a query sequence that belonged to a better sampled clade, but represented a new lineage; (c) using a sequence that represented a new variety of an already submitted species in a revised and densely sampled group. Only unpruned sequences containing also indel regions have been used for the tests.

**Figure 2:**
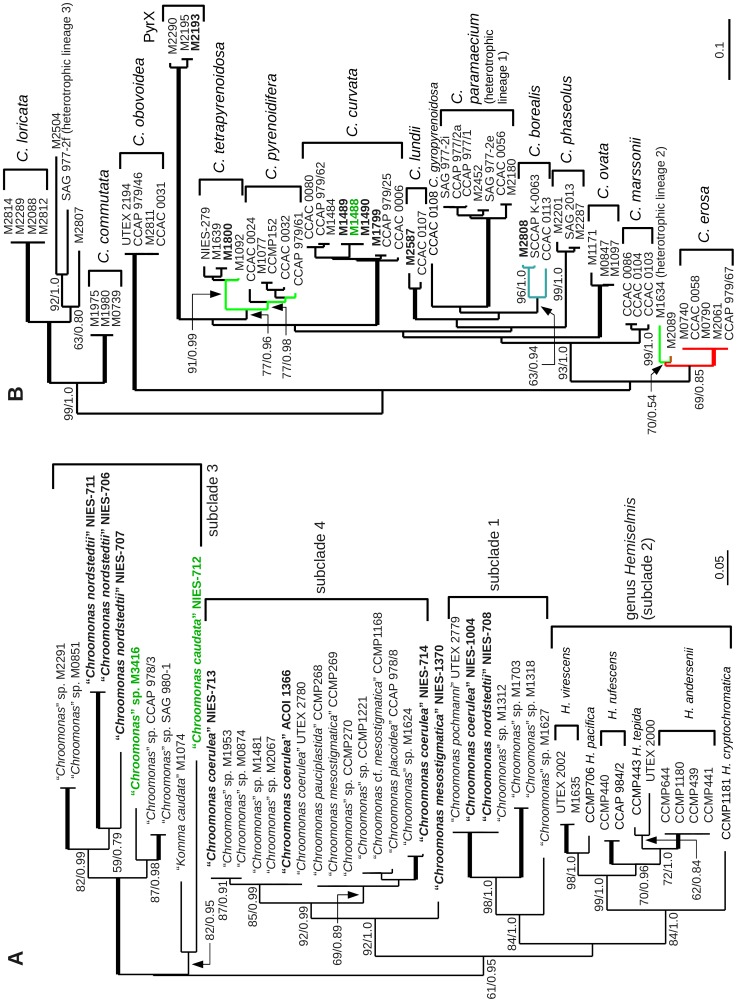
Unrooted maximum likelihood trees of partial nuclear LSU rDNA sequences of the *Chroomonas* clade and of the genus *Cryptomonas.* For both, phylogenetic tree construction and for computing distances, the same data sets with unalignable positions being pruned have been used. Evolutionary models: GTR+I+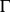; support values: maximum likelihood/posterior probabilities, support of 100%/1.0 as bold lines; scale bar  =  expected substitutions per site. **A** – The *Chroomonas* clade included three different genera. In rooted phylogenies, the genus *Chroomonas* was paraphyletic with the genus *Hemiselmis* being nested within *Chroomonas* [56]. Only the genus *Hemiselmis* has been subject to a previous revision using an integrated taxonomy approach, thus, assignment of *Hemiselmis* species corresponded to tree topology [50]. New sequences labeled in bold face, sequences used for blast queries in green (see text and Table 5). 45 taxa, 920 positions. **B** – Partial LSU rDNA phylogeny of the genus *Cryptomonas*. Species designations according to previous revisions. Turquoise branches: intraspecific distances in distance classes 4 and 5; red branches: interspecific distances in class 2, green branches: interspecific distances in class 3 (see Fig. 4 and text). 64 taxa, 975 positions; new sequences in bold face, taxon label in green: sequence has been used for blast searches (see text).

To test scenario (a), a partial LSU rDNA sequence of a not yet submitted lineage of the *Rhodomonas* clade (included also the genera *Storeatula* and *Rhinomonas*), strain CCAP 978/13, has been used as a query (1093 nt). For the other testing scenarios, newly obtained sequences of the *Chroomonas* clade (1125 and 1015 nt; Fig. 2A, OTUs labeled in green) (b) and of *Cryptomonas curvata* strains (1076 nt; Fig. 2B, OTUs labeled in green) (c) have been submitted to the blast search engines.

Different from the megablast search with a COI-5P region as a query, the default setting “megablast” yielded only cryptophyte sequences among first 100 hits under all three testing scenarios (Table 6). Similar to the tests with the COI-5P region, megablast inserted a considerable number of gaps into the pairwise alignments (Table 6). Query coverages were lower than in the megablast search with the COI-5P sequence of *C. curvata* CCAC 0080, but identities reached higher percentages, and expectancy value were zero (Table 6).

**Table 6:** Performance of megablast searches (default settings) using cryptophyte 5′-partial nuclear LSU rDNA as query sequences.

Rank	Description	Score bits	Expectancy	Identities	Gaps	Coverage
**Testing scenario (a): “** ***Chroomonas salina*** **” strain CCAP 978/13 (1093** **nt) as a query** *						
1	*Rhodomonas* sp. M1480 partial 28S rRNA gene (AM396399.1)	1652 (894)	0.0	1009/1062 (95%)	17/1062 (2%)	96%
2	*Storeatula* sp. CCMP1868 28S ribosomal RNA gene (FJ973366.1)	1459 (790)	0.0	947/1020 (93%)	24/1020 (2%)	92%
3	*Chroomonas coerulea* partial 28S rRNA gene, strain UTEX 2780 (AM901343.1)	1138 (616)	0.0	879/1001 (88%)	38/1001 (4%)	89%
**Testing scenario (b): “** ***Chroomonas caudata*** **” strain NIES-712 (1125** **nt) as a query**						
1	*Komma caudata* partial 28S rRNA gene, strain M1074(AM901329.1)	1155 (625)	0.0	909/1038 (88%)	52/1038 (5%)	91%
2	*Chroomonas* sp. CCMP 270 partial 28S rRNA gene (AM901316.1)	1107 (599)	0.0	898/1034 (87%)	54/1034 (5%)	91%
3	*Chroomonas mesostigmatica* partial 28S rRNA gene, strainCCMP 269 (AM901315.1)	1107 (599)	0.0	898/1034 (87%)	54/1034 (5%)	91%
**Testing scenario (c): ** ***Cryptomonas curvata*** ** strain M1488 (1074** **nt) as a query**						
1	*Cryptomonas curvata* 5.8S rRNA gene (partial), 28S rRNA gene (partial) and ITS2, strain CCAP 979/25 (AJ715443)	1893 (1025)	0.0	1052/1065 (99%)	2/1065 (0%)	99%
2	*Cryptomonas curvata* partial 5.8S rRNA gene, ITS2, and partial28S rRNA gene, strain CCAC 0080 (AJ566148)	1869 (1012)	0.0	1050/1067 (98%)	7/1067 (1%)	98%
3	*Cryptomonas curvata* partial 5.8S rRNA gene, ITS2, and partial28S rRNA gene, strain CCAC 0006 (AJ566147)	1869 (1012)	0.0	1048/1065 (98%)	4/1065 (0%)	98%

All other 100 first hits in testing scenario (a): cryptophyte sequences only. Only cryptophytes among first 100 hits in testing scenario (b); mixture of representatives of the *Chroomonas* clade and other genera. Only cryptophytes among first 100 hits in testing scenario (c); sequences of the genus *Cryptomonas* ranked in positions 1 to 47, followed by other cryptophyte genera.

* RW Butcher revised cryptophyte genera in 1967, but the classification resulted in an unnatural systematics [156]. Genus names thereafter have not been corrected by the culture collection.

Testing scenario (a): At time of this study, only two other sequences belonging to the *Rhodomonas* clade have been available in GenBank (“*Rhodomonas*” sp. M1480 acc. no. AM396399 and “*Storeatula*” sp. CCMP1868 acc. no. FJ973366). Both sequences appeared in positions 1 and 2, respectively (Table 6), followed by other cryptophyte genera, with a *Chroomonas* sequence first. In the alignments of the latter three, the number of inserted gaps increased from rank 1 to 3 (2 to 4%; Table 6).

Testing scenario (b): The sequence of the “*Chroomonas caudata*” strain NIES-712 represented a new, but long-branch lineage in the data set (subclade 3; see green labels in Fig. 2A). If this sequence was used as a query sequence, the next-related sequence “*Komma caudata*” strain M1074 was ranked in top position, followed by two other, but not closely related sequences of the *Chroomonas* subclade 4 (Table 6; Fig. 2A). Despite the fact that the *Chroomonas* clade was better sampled with respect to partial nuclear LSU rDNA than the Cryptophyceae as a whole with respect to COI-5P, identities did not even approach 90% (Table 6). Since megablast was supposed to function best in closely related sequences with a sequence identity higher than 95%, also discontiguous megablast and blastn have been tested. The results were worse than with megablast. Whereas the closer related “*Komma caudata*” strain M1074 remained in top rank, positions 2 and 3 were taken over by two *Cryptomonas phaseolus* sequences, both belonging to a genus not part of the *Chroomonas* clade. Due to the higher penalty of gaps in discontiguous megablast, the number of gaps was reduced from 52 to 50 in the pairwise alignment with “*Chroomonas caudata*” NIES-712, but at the same time resulted in a reduced query coverage (from 91 to 87%) and in a slight decrease of the number of identities (from 88 to 87%) (not shown). If the short-branch sequence of the newly sequenced strain M3416 was chosen as a query in megablast (1015 nt), top four ranks were occupied by its closest relatives, the strains CCAP 978/3, SAG 980–1 (both 95% max. identity), M2291 and M0851 (the two latter with 92% max. identity; Fig. 2A; blast results not shown). Similar results were obtained with discontiguous megablast.

Testing scenario (c): Concerning sampling density, the freshwater genus *Cryptomonas* represented the best-sampled group within the cryptophytes (Fig. 2B). Over 60 5′-partial nuclear LSU rDNA sequences have been available in the databases. If one of the new *C. curvata* sequences was used as a query (strain M1488, labeled in green in Fig. 1B; sequence length 1076 nt), the three search algorithms, megablast, discontiguous megablast and blastn, ranked all five published *C. curvata* sequences in positions 1 to 5 (Table 6). In the megablast results, query coverages were as high as 98 to 99%, the number of inserted gaps was lower than in testing scenarios (a) and (b) and score bits, non-normalized scores and number of identities were highest among the three testing scenarios (Table 6). Thus, 5′-partial nuclear LSU rDNA sequences in combination with megablast worked accurately even for cross-eukaryote database searches.

Both 5′-partial LSU rDNA data sets also have been tested for substitution saturation, once each separately and once as a large combined alignment (Table 4). In all three cases, the ratios of 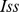 to 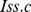 for symmetrical as well as asymmetrical trees were below 1 and the differences between 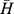 and 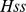 proved to be significant. Thus these data sets with a taxon sampling restricted to cryptophytes have not been saturated. The GTR+I+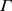 distance between the *C. curvata* strain CCAC 0080 and *C. ovata* strain NIES-274 COI-5P sequences was considerably higher than between *C. curvata* and *C. ovata* in the 5′-partial LSU rDNA data set. Partial LSU rDNA was not available for strain NIES-274, but between the three *C. ovata* strains M0847, M1097 and M1171 and *C. curvata* strain CCAC 0080, a distance of 0.090 substitutions per site has been computed for 5′-partial LSU rDNA, three-fold less than for for COI-5P with 0.293.

### Automatic Species Delimitation

#### Frequency distributions and the “barcode gap”

**Figure 3:**
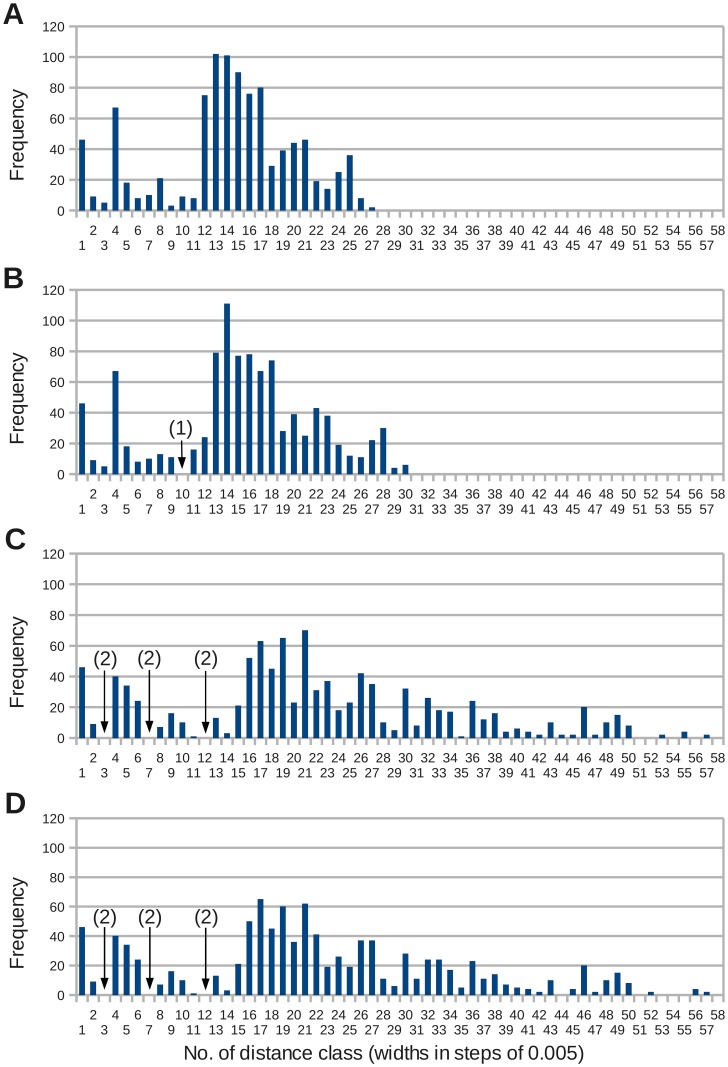
Frequency distibutions of genetic distances in the *Chroomonas* clade under p-distances and three different evolutionary models. The distances have been computed from 5′-partial nuclear LSU rDNA using the same data set as in Fig. 2A. The simpler the evolutionary model, the smaller the range of genetic distances due to an underestimation of the number of mutations. The positions of putative barcode gaps changed with evolutionary models (arrows). Sizes of the distance classes in steps of 0.005 with closed lower and open upper limits: class 1 = [0.000–0.005[, class 58 = [0.285–0.290[. **A** – The frequency distribution of p-distances does not show gaps. **B** – K2P distances with a gap in distance class 10 [0.0450–0.0500[(1). **C** – TIM2+I+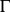. **D** – GTR+I+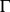. (2) Gaps in the complex evolutionary models TIM2+I+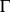 and GTR+I+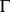 in the distance classes 3 [0.0100–0.0150[, 7 [0.0300–0.0350[and 12 [0.0550–0.0600[.

The unrooted maximum likelihood trees in Fig. 2 have been inferred from the same 5′-partial LSU rDNA sequences of the *Chroomonas* clade (Fig. 2A) and of the genus *Cryptomonas* (Fig. 2B) also used to compute genetic distances and frequency distributions (for acc. nos see File S1). Non-alignable regions have been pruned from the alignments prior to the analyses. The data set of the *Chroomonas* clade yielded an analysis alignment with n = 45 taxa, 920 positions and 
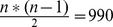
 pairwise distances (Fig. 3).

Whereas in phylogenetic analyses usually complex evolutionary models either identified by model testing as appropriate or directly the absolutely best, but most complex model GTR+I+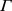 have been chosen [68], computations of distances addressing DNA barcoding mostly have relied on the simple Kimura-2-parameter (K2P or K80; [2] [69]) model or on p-distances.

Uncorrected or p-distances are computed by counting identities across two aligned sequences, subtracting the number of identities from the total alignment length and dividing this number of divergences (gaps and mismatches) by total alignment length. The resulting proportional value (“p”) multiplied by 100 corresponds to divergence in percent. Distances computed under the assumption of an evolutionary model (i.e. corrected distances) do not represent proportional values, but represent expected substitutions per site and are open to infinity. The frequency distributions in this study therefore have not been expressed in percent.

Evolutionary models consist of several parameters. Substitution rate matrices correct for different evolutionary rates in point mutations. The general time reversible model (GTR) represented the most complex rate matrix assigning to each of the six possible reversible point mutation types a different evolutionary rate [70]. All other possible reversible substitution rate matrices were special cases of the GTR model and, thus, were covered by it. The proportion of invariable sites (I) addresses conserved positions in an alignment. The among-site rate variation parameter (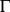) accounts for differences in mutation rates across positions of an alignment, i.e. identifies hot spots of mutation that more likely contain homoplasies [71]. The two latter parameters can be combined with any type of substitution rate matrix.

Modeltesting programs try to find a trade-off between approximating the evolutionary model fitting a data set as best as possible and complexity of evolutionary model. They search for the least complex evolutionary model that is not significantly worse than the absolutely best model. The *Chroomonas* alignment has been tested with jModeltest 0.1.1 [68]. According to the Akaike information criterion (AIC; [72]), the TIM2+I+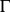 model was ranked first, followed by GTR+I+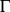, whereas K2P was the third-worst among 88 tested models. Four frequency distributions, thus, have been computed with the distance measures commonly used in DNA barcoding (p-distances and K2P; Fig. 3A and B) and with two complex models, TIM2+I+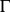 and GTR+I+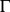 (Fig. 3C and D). The substitution rate matrices K2P and TIM2 differ in that K2P uses two different mutation rates (transitions versus transversions), whereas the transition model TIM2 differentiates between four substitution rates [68]. The two types of transitions (AG or CT), and transversions involving either an A or a G are assigned separate mutation rates, respectively. Although proportional and evolutionary distances are not directly comparable, all frequency distributions have been depicted with same scaling of axes in Fig. 3. Class widths in Fig. 3 correspond to steps of 0.005 proportional distance or expected substitutions per site (closed to the lower and open to the upper limit). To allow for a readable labeling of the abscissa, the classes have been numbered.

**Figure 4:**
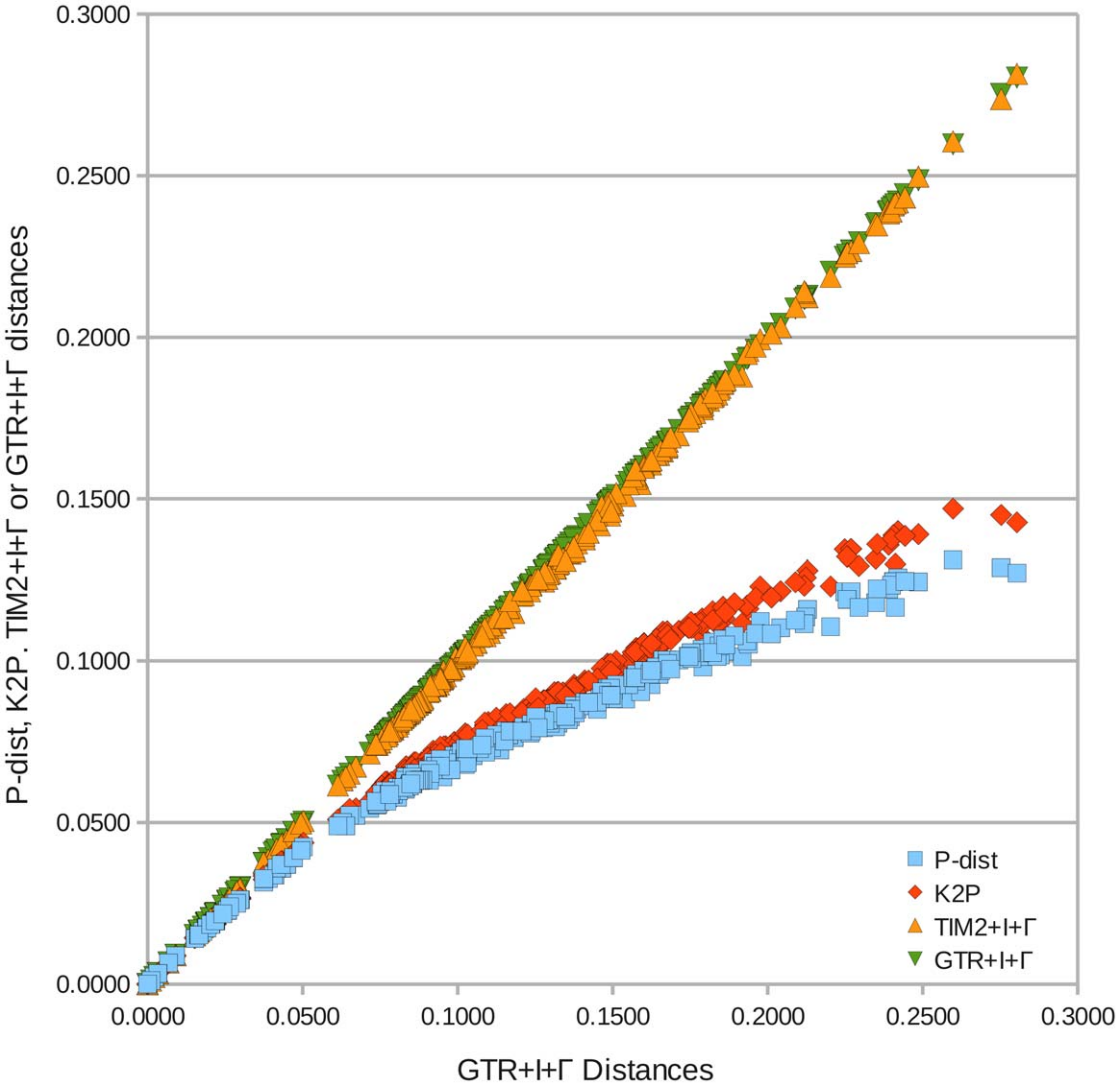
Genetic distances in the *Chroomonas* clade under different distance measures (ordinate) plotted against GTR+I+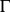 (abscissa).

The frequency distributions indicated an extreme underestimation of mutation rates in uncorrected distances and in the K2P model compared to the complex models (Figs. 3 and 4). Whereas computation resulted in genetic distances from 0.0000 up to 0.1312 and 0.1470 in uncorrected distances and K2P, respectively, the range of distance estimates almost doubled under the two complex models TIM2+I+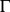 (max. distance: 0.2816) and GTR+I+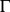 (max. distance: 0.2803) at cost of the number of counts in several classes.

All frequency distributions in Fig. 3 proved to be multimodal with peaks in classes 1 [0.000–0.0050[ and 4 [0.0200–0.0250[ and with a broader peak between distance classes 12 and 18 in p-distances and K2P (Fig. 3A and B). This peak flattened and shifted towards higher classes in the complex models (Fig. 3C and D). The shifts in distance range and in counts of distances per class also affected the position of gaps. The K2P model showed a gap in class 10 (genetic distances from 0.045 to 0.050) (Fig. 3B), whereas the distribution of gaps in the two models with gamma correction were similar in the lower distance classes (classes 3 [0.0100–0.0150[, 7 [0.0300–0.0350[ and 12 [0.0550–0.0600[) (Fig. 3C and D). A clear identification of a putative barcode gap required a gap with clear and stable slopes flanking both sides. Given the irregular shapes of the frequency distributions, however, none of gaps could serve as a barcode gap to set a threshold between intra- and interspecific distances for an identification of new species. The evolutionary model also had an effect on the ranking of the genetic distances. In uncorrected distances and in the K2P model the largest distances have been found between strain UTEX 2000 belonging to the *Hemiselmis* clade and strains NIES-706/NIES-711 (subclade 3), respectively (see Fig. 2A), whereas in both complex models the distances between strain NIES-712 on one hand side and strains NIES-706/NIES-711 on the other side were found to be largest. The two complex models resembled each other in shapes of frequency distribution and distance ranges, whereas the correcting effect of K2P versus p-distances was less pronounced (Fig. 4). Only in a small interval from 0.000 to approximately 0.02 the distances inferred from p-distance or K2P have been comparable to TIM2+I+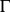 and GTR+I+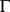, whereas the underestimation of substitution rates and, thus of potential homoplasies, became considerable in the distance classes beyond 0.050.

**Figure 5:**
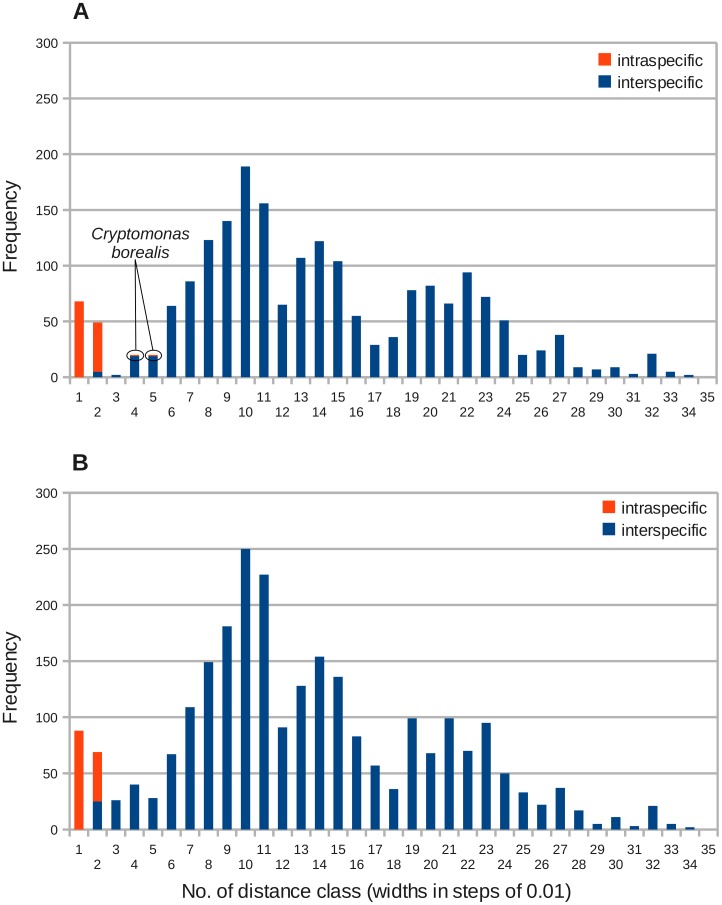
Frequency distributions of intra- and interspecific distances in the genus *Cryptomonas* in an unmodified and in a modified data set. The distances have been computed using the GTR+I+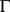 model. **A** – Frequency distribution of the unmodified data set. In *Cryptomonas borealis* taxa with long branches have been lumped to one species. Distance class 3 [0.02–0.03[contained only two genetic distances, labeled in green in Fig. 2B. **B** – Frequency distribution after duplicating the sequences of the strains M1634 and M2089 four times each to examine a hypothetically better taxon sampling in these two species. The putative blurred barcode gap filled up.

The presence of gaps not only depended in the type of the assumed evolutionary model, but also in taxon sampling. As for the *Chroomonas* data set, unalignable regions have been pruned from the alignment prior to phylogenetic analyses in the genus *Cryptomonas* (phylogeny in Fig. 2B). The final analysis alignment contained n = 64 taxa and 975 positions. To reduce the number of identical sequences predominantly found in *C. erosa* and *obovoidea*, both subclades have been restricted to 5 and 4 OTUs, respectively. The genetic distances in this data set also proved to be highly underestimated under the K2P model (largest distance: 0.145; largest distance under GTR+I+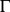: 0.333). Thus the GTR+I+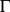 model has been used to compute 
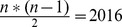
 pairwise distances (Fig. 5A). Since the genus *Cryptomonas* has been revised according to phylogeny, a consistent systematics allowed for a separation the distances into 114 intra- (red) and 1092 interspecific (blue) distances.

The frequency distribution of genetic distances in the *Cryptomonas* data set also proved to be multimodal. Except for two intraspecific distances in the classes 4 [0.030–0.040[and 5 [0.04–0.05[, all intraspecific distances were found in distances classes 1 and 2 encompassing expected mutation rates from 0.000 up to 0.02 (Fig. 5A). The two offside intraspecific distances belonged to *Cryptomonas borealis* (branches labeled in turquoise in Fig. 2B). The three strains lacked distinctive morphological characters, but displayed considerable genetic divergence, thus, likely do not belong to the same biological species. The leak of intraspecific into interspecific sequences therefore would be solved by splitting *C. borealis* into two species, with strain CCAC 0113 representing one species and the strains M2808 and SCCAP K-0063 the other (Fig. 2B). Apart from this, a minimum resembling a blurred potential barcode gap was found in distance class 3 [0.020–0.030[. The interspecific distances leaking into the intraspecific distances in distance class 2 [0.010–0.020[originated from pairwise comparisons of *C. erosa* with the strain M2089 (branches labeled in red in Fig. 2B). The two distances found in the class with minimum counts in the putative barcode gap have been computed from the two sister strains to *C. erosa*, M2089 and M1634, and from *C. pyrenoidifera* strain CCAP 979/61 and *C. tetrapyrenoidosa* strain M1092 (green branches in Fig. 2B). In both cases, it does not make sense to merge strains to eliminate this overlap. M2089, M1634 and the five *C. erosa* isolates would consist as a new “species” of photoautotrophic as well as heterotrophic members, whereas *C. pyrenoidifera* and *C. tetrapyrenoidosa* were clearly separated by genetic divergence supported by their morphology (i.e. the number of their pyrenoids).

Genetically identical isolates have been found in freshwater samples across large areas. *C. loricata* has been sampled from a river close to Cologne (strain M2287) and from a puddle close to Braunlage in the hilly area Harz (strain M2088). The distance between these two German locations comprises over 300 km. The sampling sites of the *C. loricata* were even farther apart. Most strains originated from German freshwater bodies, but one sample has been drawn from a Finnish lake (File S1). The species that participated in blurring the putative barcode gap were clearly undersampled in this data set. How does the frequency distribution reshape under an improved sampling? To simulate this situation, the sequences of the strains M2089 and M1634 have been duplicated several times to result in an artificial “M2089” clade and an “M1634” clade comprising 5 identical sequences each, corresponding to the situation in *C. erosa* in the data set. In addition the three *C. borealis* sequences have been recoded to represent two different species. The number of sequences in the alignment increased, thus, from 64 to 72 resulting in 2556 genetic distances, 132 intraspecific and 2424 interspecific ones (Fig. 5B). The gap in class 3 filled up to similar levels as the neighboring classes 4 and 5, the leak into intraspecific distances became larger and the ratio of intraspecific to interspecific distances in class 2 decreased from 8.8 (44 intra- and 5 interspecific distances) to 1.76 (44 intra- and 25 interspecific distances). Expectedly, in class 1, encompassing identical sequences, the counts increased. But the shape of the multimodal distribution also changed in classes 19 to 22 and became more irregular.

#### The general mixed Yule-coalescent model

**Figure 6:**
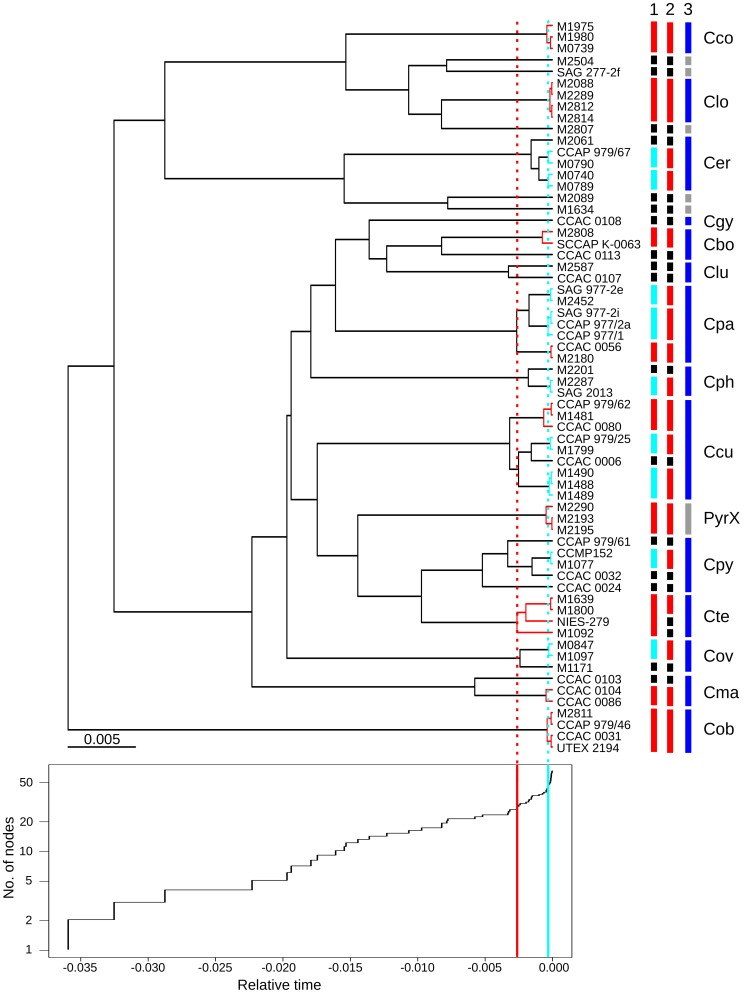
Results of the GMYC analysis: Bayesian tree inferred from the *Cryptomonas* data set under the assumption of a molecular clock (top) and the corresponding lineage-through-time (LTT) plot (bottom). Due to a lack of cryptophyte fossil record the tree could not be calibrated, thus branch lengths and abscissa of the LTT plot represent only relative time scales. The vertical red and turquoise lines in the LTT plot demarcate the two thresholds predicted by the multiple threshold model with speciation to the left and coalescence events to the right [63]. The red and turquoise branches in the tree indicate clusters of species identified by the two thresholds, respectively. The bars to the right of the tree’s terminal nodes represent the species predicted by the multiple threshold GMYC model (1), by GMYC with a single threshold (2) and according to previous revisions using a combination of multiple molecular markers and morphology [33] [20]. Black bars represent singletons, gray bars unrevised putative species. Clade PyrX has not been merged to one species in the previous revisions due to considerable divergence in internal transcribed spacers 2, whereas the single strain of *Cryptomonas gyropyrenoidosa* has been described due to a unique set of morphological characters. The ordinate of the LTT plot has been logarithmized. 0.000 in the abscissa represents present. Cbo, *Cryptomonas borealis*; Cco, *Cryptomonas commutata*; Ccu, *Cryptomonas curvata*; Cer,*Cryptomonas erosa*; Cgy, *Cryptomonas gyropyrenoidosa*; Clo, *Cryptomonas loricata*; Clu, *Cryptomonas lundii*; Cma, *Cryptomonas marssonii*; Cob, *Cryptomonas obovoidea*; Cov, *Cryptomonas ovata*; Cpa, *Cryptomonas paramaecium*; Cph, *Cryptomonas phaseolus*; Cpy, *Cryptomonas pyrenoidifera*; Cte, *Cryptomonas tetrapyrenoidosa.*

The Bayesian and subsequent GMYC analyses with the *Cryptomonas* data set yielded the ultrametric tree and the lineage-through-time plot depicted in Fig. 6. The tree inferred under the assumption of a random local clock has been imported also into Paup to compute likelihood scores for an unconstrained tree and for evolution under the constraint of a strict molecular clock. The null hypothesis of a strict clock was rejected in a likelihood ratio test with P = 0.000. Also the likelihood ratio tests of coalescence only versus the mixed Yule-coalescent models resulted in rejections of the null hypotheses (coalescence only) with P<0.0001 (for both GMYC models, multiple and single threshold). The authors of the R script for performing GMYC analyses caution to use the multiple threshold model, since it was still in beta test phase and would take a long time for computation (see GMYC help text in R) [63] [73]. For the *Cryptomonas* data set with 64 sequences, however, computation succeeded within few minutes. The GMYC model accounting for multiple thresholds identified two different switch positions, one at −0.0026 (red line in the LTT plot in Fig. 6) and one at −0.0003 (turquoise line) relative time units into the past (Fig. 6, bottom), whereas in the single threshold model the switch was found at −0.0007 (not shown). The difference in likelihoods between the two models, however, proved to be not significant (P = 0.553) and both models also yielded similar results (bars to the right of the tree in Fig. 6). Multiple as well as single threshold model identified 18 clusters (i.e. separate populations; confidence interval of multiple threshold model: 17–19; confidence interval of single threshold model: 16–18). Each of the two thresholds in the multiple threshold model identified nine clusters, respectively. The two models slightly differed in the absolute number of entities: 35 in the multiple threshold and 37 in the single threshold model (Fig. 6). The difference in counts was caused by a different treatment of *C. tetrapyrenoidosa* (Cte), which was considered one species under the multiple threshold model, whereas it was split into one cluster and two singleton lineages under the single threshold model (Cte in Fig. 6).

Both models, however, predicted more species than have been erected in previous revisions based on a combination of molecular markers and morphology (14 clusters and 6 singletons  = 20 entities; Fig. 6). All of the species clusters, except for the unrevised clade PyrX, *C. commutata* (Cco), *C. loricata* (Clo) and *C. obovoidea* (Cob), have been subdivided into smaller clusters and singletons by the GMYC analyses. Four species consisted exclusively of identical sequences: *C. commutata* (Cco; 3 sequences), *C. erosa* (Cer; 5 sequences), *C. loricata* (Clo; 4 sequences) and *C. obovoidea* (Cob; 4 sequences) (Fig. 2B). One would expect to find all identical sequences in one GMYC cluster and this proved to be true for *C. commutata*, *C. loricata* and *C. obovoidea*. However, the five identical sequences of *C. erosa* surprisingly have been split into two clusters and one singleton by both GMYC models, corresponding to three separate species (Fig. 6). The underlying ultrametric tree displayed considerable differences in branch lengths in the latter species, although no sequence divergence was present among the strains. Additional experiments with ultrametric trees derived under other clock model settings did not really solve the problem. In most cases, *C. erosa* has been separated into three species. The best results have been obtained with the – rejected – strict clock model and the single threshold GMYC model. All four species were predicted properly, likely because intraspecific branch length variation in clades with identical sequences was lowest among all generated trees, but at the cost of lowest number of clusters (14) and highest number of singletons (21). In this tree no intraspecific variation was accepted by the GMYC model. The predicted species clusters contained only identical sequences. The same tree, however, yielded the worst results under the multiple threshold model, which identified 4 thresholds (not shown). All species with more than three identical sequences have been split up into two species or more (*C. erosa*, *C. loricata* and *C. obovoidea*), whereas at the same time highly divergent clades became merged to one species, *C. borealis* and *C. lundii* on one hand side as well as *C. ovata* and *C. marsonii* on the other side (not shown).

## Discussion

### Species Identification

DNA barcoding can be used for two tasks that differ considerably in complexity: (1) For an accurate identification of already characterized species using a reference database and (2) for an automatic delimitation of species that allows also for an identification of new species. The latter of the two is more challenging, but considered inevitable to cope with the masses of sequence data derived from environmental DNA in combination with next generation sequencing (“DNA metabarcoding”) [74]–[75].

The advantages of DNA barcoding for protists are obvious. Instead of time-consuming light microscopical observations of Lugol-fixed cells using misleading morphological characters, species diversity in a habitat could be assessed by PCR-amplifying barcode markers from environmental DNA and by subjecting the PCR products to next generation sequencing techniques. This would allow for a rapid identification of species at large quantities within a short time. Different from previous diversity assays using nuclear SSU rDNA sequences [26]–[27], however, DNA barcoding aims at correctly identifying the presumed smallest units of evolution, species [2]. Monitoring of the performance of a candidate barcode marker by morphology often is not possible in protistan lineages due to their small sizes and due to a lack of distinctive characters. Therefore testing of a candidate barcode marker requires a culture collection covering the diversity of a group as best as possible, time-consuming mating experiments to identify biological species or an alternative species concept in asexually reproducing lineages, and phylogenetic analyses to gain an *a priori* knowledge of the presumably correct phylogeny of the group [76]–[77]. It is, thus, tempting to skip this tedious testing phase by using an algorithmic species delimitation method. Instead of performing morphological examinations one could use only a short highly variable DNA barcode marker for assessing diversity and for delimitation and identification of new species at once.

Task (1), an accurate identification of species, in first place requires a well-sampled database with high quality reference sequences and a pre-defined and consistent systematics down to species level [8]. NCBI’s blast suite has been designed for finding identities between pairs of sequences, yielding a hit list of identical to similar sequences [64] [78]. The scores generated from awards and penalties represent unproportional and uncorrected distances [64]. This simple evaluation system may easily fail to identify close relatives due to an underestimation of substitution rates and due to substitution saturation [79]. In the case of the cryptophyte COI-5P sequence, this vulnerability of the blast tools has been drastically demonstrated. Since no cryptophyte sequences showed up under the first 100 hits, the megablast search was repeated constrained to cryptomonads. Constraining searches to a group has been recommended to reduce the risk of misidentifications in non-identical sequences [64]. This approach, however, yielded no hits at all. Discontiguous megablast and blastn at least identified the *Cryptomonas curvata* query as most closely related to the only congeneric sequence in the databases, *Cryptomonas ovata*, albeit with only 83% identity. The search results were not convincing, though. Only one of the three other cryptophyte sequences was found among first 100 hits in position 24, and the remaining two cryptophyte *cox*1 sequences were found far below. Peculiarly, the COI-5P sequences following the top hit *C. ovata* belonged to the giant kelp *Macrocystis pyrifera*. The Cryptophyta and the stramenopiles, to which the brown algae belong, represent two different phyla in the eukaryotic tree of life [24]–[25]. An experienced blast user without organismic knowledge could have interpreted the only correct hit in top position as a contaminant, since almost all other sequences belonged to stramenopiles.

In such an extremely badly sampled “reference database” with only four cryptophyte sequences not only the restricted length of COI-5P may have resulted in megablast failing to identify the query sequence as a cryptophyte. In protein-coding genes, 3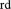 codon positions evolve at higher rates than 1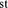 and 2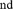 codon positions. The test for substitution saturation in COI-5P indicated that saturation of 3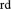 may be one explanation for the misleading results of the blast searches. The high mutation rates probably have resulted in background noise blurring information about relatedness. Since megablast requires an exact match across 28 nucleotides as a seed to start searches with, this may have accounted for its complete failure. A word length of 28 nt requires 9 exactly matching codons in a stretch. In discontiguous megablast and blastn, seeds with a length of only 11 nucleotides seemingly were sufficiently short to succeed in identifying the only congeneric sequence *C. ovata* as the next relative to the query *C. curvata*. Despite of a strong bias towards animal COI-5P sequences in the databases, brown algal and oomycete sequences prevailed among the megablast, blastn and discontiguous megablast hits (keyword searches in Entrez with the strings “eukaryotes AND COI” and “Metazoa AND COI” resulted in roughly 780,000 and 764,000 hits, respectively, whereas a search addressing “stramenopiles AND COI” yielded only 3,500 hits predominantly with plant-pathogenic oomycetes and brown algae, both stramenopile lineages). Similarities in codon usage have been found e.g. to result in artifactual phylogenetic trees in dinoflagellates [15]. Biased codon preferences may also be causative for the search results with COI-5P, since different from the majority of barcoded animals, stramenopiles and cryptophytes use the standard code for mitochondrial genes, reported for the cryptophyte *Hemiselmis andersenii*, the brown alga *Laminaria digitata*, and the raphidophyte algae *Heterosigma akashiwo* and *Chattonella marina* [80]–[82]. Also potential sequencing errors or alignment artifacts resulting in gaps may have severe impact on the ranking positions of protein-coding sequences that are highly conserved at protein level. COI-5P sequences can be aligned without gaps across cryptophytes and stramenopiles, thus insertion of only one gap increases the scores considerably. Editing the COI-5P sequence of *C. curvata* in only two positions to avoid two gaps in the pairwise blast alignment with the sequence of the cryptophyte *Guillardia theta* moved the latter from ranking position 24 up to 5.

Queries with 5′-partial nuclear LSU rDNA proved to be more robust. Concerning the “*Chroomonas (Rhodomonas) salina*” strain CCAP 979/13 sequence, taxon sampling of close relatives was similarly bad to COI-5P in the genus *Cryptomonas*. Although only two other sequences of the same clade have been available in the databases, these two were ranked as first hits and other farther related cryptophytes were found below in megablast searches. Identification success of megablast, thus, proved to be also dependent in the type of query DNA. In better sampled groups, megablast safely identified the closest relatives of the respective query sequence, in the *Chroomonas* clade as well as in the genus *Cryptomonas*. The testing scenarios encompassed different classification levels, cross-genus comparisons with the “*Chroomonas (Rhodomonas) salina*” strain CCAP 979/13 sequence, congeneric with the *Chroomonas* sequences and intra-specific in the genus *Cryptomonas*. Ribosomal RNA genes consist of conserved regions alternating with indel-rich highly variable regions that also complicate alignment [83]. The conserved helical domains probably facilitated finding of 28 nt long seeds by megablast, whereas the naturally occuring indels resulted in high gap costs, but at the same time may also have enhanced resolution. Possibly the identification success of megablast with 5′-partial nuclear LSU rDNA was not only related to the structure of rRNA genes. On one hand side, the 5′-partial nuclear LSU rDNA sequence of “*Chroomonas (Rhodomonas) salina*” strain CCAP 979/13 was almost twice as long as the COI-5P sequence of the *C. curvata* strain CCAC 0080, on the other hand identities between the query sequence and the two top hits were higher in the RNA gene (95 and 93%, respectively) and its evolutionary rate at least in *C. curvata*/*C. ovata* comparisons was three times less. Candidate barcode markers have been shown to differ in evolutionary rates and probably this is true also for ribosomal versus mitochondrial protein genes in cryptophytes. Zhao et al. examined several potential barcode markers of similar lengths in fungi (around 400 to 500 nucleotides) [84]. The two protein-coding genes 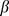-tubulin and EF-1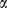 had higher evolutionary rates especially in interspecific comparisons than the two ribosomal DNA regions ITS and partial LSU rDNA. Future research in cryptophytes will also require such comparative studies to assess resolution of different potential barcode markers to determine the best-suited ones.

The results of this study underline the importance of a well-sampled reference database for an accurate identification of species. The better sampled a database was, the more accurately identification worked. The increase in accuracy could be observed in all types of nucleotide versus nucleotide blast tools. It can be assumed also for COI-5P that blast searches will become more reliable once a reasonable reference database has been set up with a similarly dense sampling as the partial nuclear LSU rDNA sequences in the genus *Cryptomonas*. In such a database, the vulnerability of blast may even become irrelevant, since an accurate identification of species relies on the presence of identical conspecific reference sequences in the database. The simplest and most primitive clustering algorithm as well as blast may, thus, do. To work reliably, however, the reference sequences need to be of high quality. Low quality reference sequences may cause severe setbacks in protistan research, because they pass on in a snowball system of biased results [47] [85]. The latter cautions against using next generation sequencing techniques instead of double-stranded Sanger sequencing especially for generating reference sequences [47] [85]. Also PCR and subsequent cloning may reduce the quality of reference sequences [47]. Although ribosomal DNA such as the LSU rDNA fragment used in this study seem to be less vulnerable, a high quality of the reference database should be self-evident for all chosen barcode markers.

In practice, erroneous identifications as observed for the COI-5P sequence in this study most likely occur in blast searches performed to rule out contaminants or unspecific PCR amplifications in badly sampled groups. Misidentifications also have to be expected during test runs in the process of establishing a reference database, but may as well affect the assignment of environmental sequences to higher classification levels. In such cases using a translated COI-5P sequence instead could be an option to rule out severe misidentifications. Blast searches with protein (blastp) or translated nucleotide (blastx) queries have been usually preferred over nucleotide searches to circumvent issues with substitution saturation, codon usage or compositional biases [86]. A higher similarity due to a higher conservation at amino acid level and due to better search algorithms based on pre-defined substitution scoring matrices such as BLOSUM62, thus, could result in a better performance. The blastx results in this study were better than in the search with nt versus nt searches, since all cryptophyte sequences have been found among first 100 hits. But surprisingly not even a blastx search resulted in a ranking of all cryptophytes in top positions 1 to 4. Anderson and Brass reported a bad performance of all tested search algorithms if the number of amino acids was below 200 [86]. The latter was true for the *cox*1 fragment used in this study. A nucleotide sequence of 570 nt yields a protein sequence of only 190 amino acids. Such blastx search results, thus, have to be considered with great caution. For a safe identification at higher classification levels protein sequences more than 200 amino acids are required.

Except for the search tools of the blast suite (INSDC databases) or searches with profile hidden Markov models (BOLD*Systems*), other methods of species identification have not been implemented in these public databases. The search algorithm of the Barcode of Life Database, BOLD*Systems*, combines blast with profile hidden Markov models, that are supposed to outperform the blast algorithms [16] [87]. HMM software has to be trained on an alignment of the respective gene. The resulting HMM profile is based on probabilites for matches, mismatches and transitions between character states and can be used to identify similar sequences from a database. A detailed description of the search algorithm implemented in BOLD*Systems* has not been published to date. As of March 2012, the identification engine allowed only for a choice between animals, fungi and embryophyte plants using the barcode markers COI-5P, ITS, and *rbc*L/*mat*K, respectively. Several projects addressing protistan lineages have been started, but were not accessible for searches with a query sequence. This refers also to a COI-5P project “Cryptophyte culture collection barcodes” (CPTO). Thus, the performance of BOLD*Systems*’ identification engine could not be tested with the COI-5P sequence of *Cryptomonas curvata*.

Most other methods of species identification rely on sequence similarity and may be distance- or character-based in combination with or without phylogenetic tree inference [74]–[75] [88]. The most straightforward possibility to identify a species using a local data set is phylogenetic analysis. The new sequences are added to an alignment that represents the reference database followed by tree inference. In this study maximum likelihood trees have been computed under the GTR+I+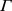 model for a direct comparison with the blast search results. The strains M1488, M1489, M1490 and M1799 have been identified as *Cryptomonas curvata*, because they were nested in the highly supported *Cryptomonas curvata* clade. If a phylogenetic tree is used only to assign a species name to a sequence by clustering, i.e. to identify it by its grouping with identical or almost identical conspecific sequences without considering deeper nodes of a tree, also an analysis with a simple evolutionary model probably will yield accurate results. In most previous studies species identification has been performed with the faster distance-based neighbor-joining algorithm in combination with the simple K2P model, e.g. [89]–[90]. K2P is a special case of the GTR+I+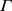 model. If K2P approximates molecular evolution in a data set, its distance measures will be comparable to those computed under GTR+I+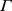. Under the latter model the distance ranges in the *Chroomonas* and *Cryptomonas* data sets, however, almost doubled, indicating an extreme underestimation of mutation rates by K2P. Plotting all computed distances measures of the *Chroomonas* data set against GTR+I+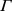, demonstrated that the simple distance measures started diverging from the two complex models already at genetic distances as low as 0.02 expected substitutions per site. The distance range of the 5′-partial nuclear LSU rDNA sequences in the genus *Cryptomonas* was even larger than in the *Chroomonas* clade. Thus, already in intrageneric comparisons the genetic distances exceeded considerably the threshold for which K2P or p-distances can be expected to be congruent with those of the complex models. Consequently, cross-genus, -family or -order comparisons under a simple evolutionary model will likely result in extremely biased distance estimates and, thus, can not be trusted [11]–[13]. In pairwise comparisons of *Cryptomonas curvata* versus *Cryptomonas ovata* COI-5P sequences, distance measures under GTR+I+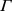 were three-fold higher (0.293 expected substitutions per site) than for the same combination of species with different *C. ovata* strains in 5′-partial LSU rDNA (0.090), indicating a higher variability of COI-5P compared to partial nuclear LSU rDNA and as a consequence that the underestimation of substitution rates by K2P may be even more severe.

Similar to an identification by phylogenetic trees most other proposed species identification methods not implemented in the public databases usually require setting up a suitable bioinformatics pipeline on a local computer, i.e. the installation of the respective software tools and of the reference databases (e.g. as an alignment). To circumvent some of the shortcomings of blast, its ignorance of phylogenetic and taxonomic context, but also to avoid the permanent maintenance of large alignments as reference databases on a local computer, Munch et al. introduced an automated character-based species identification method that included inference of phylogenetic trees [91]. The method uses Bayesian statistics with Markov chain Monte Carlo sampling to compute posterior probabilities for an affiliation of the new sequence to a clade [91]. Blast is used to retrieve sequences from NCBI’s nucleotide database. Also the corresponding taxonomic information for each sequences is obtained from the taxonomy browser of NCBI. By a heuristic sampling strategy, that also includes cut-offs for blast scores, the number of sequences is restricted to facilitate computation within a reasonable time, but with optimal coverage of species diversity and under consideration of higher rank classification. The sequences are aligned with the query sequence by ClustalW and subjected to a Bayesian analysis. The program generates a vector graphics as an output with a ladderized tree showing all higher rank taxonomic affiliations down to species with posterior probabilities for each assignment. The performance of the software obviously depends in the taxonomic annotation of the NCBI taxonomy browser. As the authors pointed out, identification will be inconsistent in groups with an unnatural systematics [92]. At time this study has been conducted, cryptophyte classification in NCBI’s taxonomy browser severly deviated from phylogeny. Some plastid-bearing cryptophytes have been merged with the phagotrophic and aplastidic genus *Goniomonas* into one order Cryptomonadales, whereas related taxa have been separated. The genus *Hemiselmis* and its synonym *Plagiomonas* have been assigned to two different orders, similarly *Plagioselmis* and its – presumably – alternative life stages *Teleaulax* and *Geminigera* [93]–[95]. Under such circumstances it cannot be expected that the method by Munch et al. will work reliably.

Species identification methods will work accurately only in groups with known taxonomic context and with an appropriate representation of species diversity in the reference database [8] [88] [91]–[92]. If a query sequence is found outside of reference clades, e.g. as a sister to a known species, the sequence may belong to the closely related species, but may as well represent a new species [88] [96]. Sequences that can not be safely assigned to a clade require further research [96].

### Species Delimitation

The masses of sequences generated from environmental DNA in combination with next generation sequencing resulted in taxonomy to lag behind considerably especially in microscopic life [48]. In addition, many new evolutionary lineages have been found that could not be assigned even to higher classification ranks, not to mention species, e.g. [26]–[27]. Thus, the option of delimiting species – including the identification new species – without prior knowledge of taxonomy, was brought into focus as a potential application in DNA barcoding. The computation of a frequency distribution based on genetic distances with a subsequent search for a barcode gap to differentiate between intra- and interspecific distances probably represented the oldest and simplest method [2] [97].

A barcode gap formed by nature during speciation processes should become obvious also in frequency distributions of genetic distances without distinguishing between intra- and interspecific distances. In this study, frequency distributions have been generated from two different data sets. In the *Chroomonas* data set, systematics was still in a state of mess, thus, its frequency distribution could be examined without being prejudiced by the idea of species limits. The revised genus *Cryptomonas* provided a data set with a consistent systematics at species level, that could be used to differentiate between intra- and interspecific distances. The frequency distributions inferred from the two data sets proved to be highly volatile in shapes depending in evolutionary models and in taxon sampling. No matter, which evolutionary model has been chosen, the *Chroomonas* data set yielded frequency distributions with irregular and multimodal shapes. Neither were the positions of gaps stable nor was an obvious “barcode gap” discernible.

Different from the *Chroomonas* data set, a minimum at distances between 0.0200 and 0.0299 in the frequency distribution of the *Cryptomonas* data set resembled a blurred barcode gap separating most intra- and interspecific distances from each other. Two astray intraspecific distances in the frequency distribution of interspecific distances have been caused by a too wide species definition of *Cryptomonas borealis* and could be eliminated in a future revision by splitting the species into two. The latter would be reasonable since the two lineages probably were genetically too divergent to be interbreedable. The putative “barcode gap”, however, proved to be an artifact caused caused by unequal taxon sampling. Two distances were present in the gap, one inferred from a comparison of strains M1634 and M2089, the other from a comparison of *C. curvata* strain CCAP 979/61 with *C. tetrapyrenoidosa* strain M1092. Since no other representatives of these species were available, a better taxon sampling was simulated by duplicating the sequences of strains M1634 and M2089 several times. Considering that most of the intraspecific distances in this frequency distribution belonged to distance class 1 with genetic distances between 0.000 and 0.010, the simulation situation represented a likely scenario of a better taxon sampling. As a result, the “barcode gap” filled up. In a massively extended data set it can be expected that most of the gaps will disappear under reshaping of the frequency distribution with positional changes of minima and maxima.

Overlaps in intra- and interspecific frequency distributions have been critically discussed in several metazoan taxa, e.g. in [8] [90] [98]–[99]. Wiemers and Fiedler examined COI-5P frequency distributions in butterflies and came to the conclusion that the barcode gap was an artifact of insufficient taxon sampling [100], whereas Little and Stevenson used a hypothetical alignment of four sequences to explain why fixed thresholds of sequence divergence in distance-based analysis do not work [88]. These results were not surprising from an evolutionary point of view. Only in a data set with strict clock-like evolutionary rates and with speciation events synchronized in time across all lineages a real barcode gap would form. Development of species, however, is influenced by many environmental factors such as geography, climate or intra- and interspecific interactions, resulting in irregular patterns of speciation events within a genus [101]–[102]. Also time intervals required for completion of speciation differ across lineages. Whereas some species may have been reproductively separated, other species within the same genus may still be able to hybridize [101]–[102]. The changes in selective pressures are also reflected at molecular level. A lack of recombination, genetic bottlenecks or changes in mode of nutrition, e.g. from photoautotrophy to heterotrophy or from free life to parasitism, correlated with accelerated evolutionary rates in genes or complete genomes [103]–[105]. Loss of photosynthesis has been found to be accompanied by massive losses of photosynthesis-related genes [106]–[108], whereas in retained genes, e.g. in *rbc*L, relaxed selective constraints have resulted in increased substitution rates [109]–[110]. Unequal evolutionary rates and base composition biases have been found also in mitochondrial genomes, thus likely affect also COI-5P [111]. Substitution rates of mammalian mitochondrial genes e.g. depended in position of a gene relative to the origin of replication [112].

Despite of these findings, sometimes blurred, but nevertheless pronounced barcode gaps have been reported e.g. from birds, moths and bivalves [113]–[115]. This may be related to the use of mean interspecific distances for plotting frequency distributions. Meier et al. pointed out that the use of mean instead of smallest interspecific distances results in an exaggeration of the barcode gap [58]. In a publication addressing DNA barcoding in anthozoans, the McFadden et al. followed the recommendations of Meier et al. and plotted frequency distributions of largest intraspecific versus smallest interspecific distances [99]. As a result not even a faint barcode gap was observable. Also in a study addressing intraspecific, congeneric and confamilial frequency distributions of COI-5P in birds and fishes, no barcode gap has been discernible [116]. Not only from the point of view of creating an artifactual “barcode gap” computation of mean values and of standard deviations seems unreasonable. In the *Cryptomonas* data set only congeneric sequences have been examined. The frequency distribution derived from the total number of interspecific comparisons was multimodal with three major peaks, whereas the asymmetric intraspecific frequency distribution had highest counts in identical sequences. High counts for identical sequences in single species were rather the rule than the exception in this study and have been observed also in animals, e.g. in fishes and birds [116]. Under such conditions, mean values will be extremely biased and may even result in negative values for one- or two-fold standard deviations below the computed mean in intraspecific distances. Population geneticists preferred mismatch distributions inferred from absolute counts of divergent sites between pairs of sequences to examine the genetic structure of panmictic populations. In a simulation study addressing non-recombinant mitochondrial genes in stagnating and in exponentially growing populations, the shape of mismatch distributions depended in the current status of a population and displayed a chaotic pattern [117]. Populations in stationary phase resulted in highly irregular multimodal mismatch distributions that differed between each replication of the simulation. Only in exponentially growing populations, mismatch distributions approximated Poisson distributions as would be expected in a random process with discrete characters.

An automatic delimitation of species by fixed thresholds of genetic divergence became more and more doubtful, resulting in proposals of alternative methods. Protistan researchers, however, are confronted with some problems when trying to apply these methods, since they usually have been established to delimit metazoan species. Species delimitation methods can be roughly subdivided into methods that use discrete characters as diagnostic features and phylogeny-based methods [59] [118]–[119]. Most species delimitation methods require *a priori* knowledge about biogeography, that proved to be difficult to predict even in multicellular organisms [59]. Protists do not necessarily show a biogeographical distribution. Planktonic organisms drift in the oceans’ streams and even freshwater species may be globally distributed. The smaller the cell size of a species, the more likely it spreads globally [60]. Fenchel and Finlay based their hypothesis of cosmopolitan distribution in microscopic life on the misleading morphospecies concept. However, environmental DNA not only unveiled an unexpected protistan diversity and potential endemic species, but also that the notion of cosmopolitan distribution nevertheless may be true for many protistan lineages [61] [120].

Pons et al. introduced as a method for species delimitation the general mixed Yule-coalescent (GMYC) model and pointed out as one of its advantages that it required no priorily defined biogeographical distributions of populations [59], thus this method could have offered a solution to the problem of species delimitation in protistan lineages. The improved GMYC model invented by Monaghan et al. used trees inferred under the assumption of a relaxed uncorrelated clock and accounted for differences in speciation/coalescence events across lineages by multiple transition thresholds to predict species clusters [63]. Usually, calibrated ultrametric trees have been used for GMYC to obtain absolute branching times for the species. Only protistan lineages producing sufficient inorganic material at least during some stages of their life history can be found in a fossilized form, e.g. diatoms (silica frustrules), foraminifers (calcite, aragonite or silica cell walls), haptophyte coccolithophorids (calcium carbonate scales), chrysophytes or testate amoebae (silica scales) [23]. For cryptophytes, as for most protists, however, no fossil record was available that could be used to calibrate an ultrametric tree. Therefore the analyses have been performed without dating information resulting in a scaling of unspecified relative time units. Also, the least constraining clock setting, the random local clock has been chosen for inferring the ultrametric tree. According to Drummond and Suchard all other clock models were special cases of the random local clock, which, thus, accounted for all of them [121]. The R scripts used by Monaghan et al. for computing the GMYC models have been implemented in the R package SPLITS, but according to the supplied help text the multiple threshold model was still in beta test phase [63] [73]. Thus, both GMYC models, the presumably more stable single threshold as well as the potentially less reliable but putatively more appropriate multiple threshold model, have been tested in this study using the *Cryptomonas* data set.

At first site the results seemed reasonable with species predictions of single and multiple threshold models being largely congruent, although the multiple threshold model differentiated between species under two transition thresholds. The only difference was found in *Cryptomonas tetrapyrenoidosa*, which was split into three species under the single, but merged to one species under the multiple threshold model. Previous species delimitations using phylogenetic information and ITS2 secondary structure in combination with morphology, resulted in 14 described species, one of which was a singleton with distinctive morphology, whereas 5 strains had not been assigned a species name, since as singletons they did not allow for an identification of synapomorphies or unique combinations of molecular characters, but also displayed no distinctive morphological characters [20] [33]. Together with clade PyrX, which was considered to consist of 2 putative species due to too variable ITS2 regions, 21 species have been identified [20]. Under the GMYC models, however, most of these species have been split up, increasing the number of putative species in the *Cryptomonas* data set to 35 (multiple threshold) and 37 (single threshold model), respectively. For those species that displayed some intraspecific variation, such as *C. curvata*, *C. pyrenoidifera* or *C. paramaecium*, a decision for splitting up did not seem implausible. Only few *Cryptomonas* species can be identified solely by morphology (*C. gyropyrenoidosa*, *C. ovata* and at least by their campylomorph morphotype also *C. curvata* and *C. pyrenoidifera*) [33]. Splitting the latter three into several species would further complicate species identification in *Cryptomonas*, but would be required, if these really represented separate biological species. However, the observation that a clade consisting of identical sequences only was predicted to comprise three biological species casted doubts on the reliability of the GMYC species predictions in this data set. In most cases the clade with the highest number of identical sequences was affected (*C. erosa* with 5 strains), whereas the species with the lowest number of sequences was never subject to this artifact (*C. commutata* with 3 sequences). The GMYC model included likelihood ratio tests that required trees to be strictly bifurcating. Forcing a tree to be dichotomous poses constraints on tree inference. For the dichotomous trees BEAST had to compute divergence times and to assign branch lengths to each terminal node also in clades with identical sequences. In *C. erosa* this resulted in a relatively early coalescence possibly overlapping with speciation in other lineages assigned to the same transition threshold. The problem probably increases with the number of identical sequences in a clade and was present in both, multiple and single threshold models. The only correct prediction of species with identical sequences was achieved in a tree inferred under the strict clock, which has been rejected in a likelihood ratio test, and in combination with the single threshold model assuming a synchronized transition from speciation to coalescence. Under this threshold, however, no intraspecific variation at all was accepted and only identical sequences were predicted to belong to one species. For the same tree, extreme artifacts were found in species delimitation under the multiple threshold model. Under the four putative transition thresholds, three of four species with identical sequences have been split up into several species and twice two genetically highly divergent species were merged into one. In the latter setting, the combination of an inappropriate clock model with the possibly more vulnerable multiple threshold GMYC model in beta test phase may have contributed to these drastic effects.

The GMYC model has been increasingly used for species delimitation in diverse animal groups, but has been reported to work also in bacteria, fungi, or macroalgae [122]–[124]. Lohse demonstrated in simulation studies, that the GMYC model highly depended in taxon sampling and in population structure, i.e. undersampling caused biased numbers of clusters [125]. Papadopoulou et al. responded to the criticizms raised by Lohse pointing out that GMYC predictions were hypotheses and every method would be sensitive to undersampling, which likely is true [126]. To identify erroneous results in a GMYC analysis, they considered a plausibility control of the output necessary by comparing the GMYC results with biogeographical distribution and morphology, and by using multiple barcode markers. However, a not required *a priori* knowledge of biogeography was originally mentioned by Pons et al. as one of the advantages of their method [59], which was the rationale for testing the GMYC model for species delimitation in protistan lineages.

What was the situation in the *Cryptomonas* data set used in this study? (1) The taxon sampling was not optimal for a GMYC analysis: Individual species were highly unequally sampled, singletons were present and some species did not display any intraspecific variation. (2) The species were neither biogeographically distributed nor did they represent populations. The *C. obovoidea* strains M2811 and CCAC 0031 e.g. have been isolated from German lakes, whereas the strain UTEX 2194 originated from a lake in the USA. The *C. erosa* strains derived from different lakes in Germany, Austria and Finland. The situation was similar in species that displayed a higher intraspecific variability. *C. curvata* strains have been retrieved from different lakes in UK and Germany, *C. pyrenoidifera* strains from Australia, Germany, Czech Republic and UK. All strains represented clones of individual cells, in most cases isolated by the capillary method. (3) Many species can not be safely identified by morphology due to a lack of distinctive characters (e.g. *C. commutata*, *C. erosa*, *C. phaseolus* and *C. obovoidea*).

It may be possible that some of the weird results (i.e. the splitting of identical sequences into several species) were artifacts caused by an unequal taxon sampling, but it is also clear that the plausibility controls, Papadopoulou et al. considered necessary to rule out false clustering by the GMYC method, could not be applied to this data set [126]. Also, achieving a good sampling of species is generally difficult in protistan lineages. Rather than being biogeographically distributed, many planktonic species show temporal patterns of distribution, e.g. in dimictic lakes [127]–[129]. Different species competing for the same resources may be found at different times in a habitat, following a chaotic oscillation pattern [130]. Thus, if DNA samples are drawn from lakes for an environmental sequencing project, they may not reflect the species diversity in the respective habitats. Species that reside as resting stages in the mudd bloom perhaps in one of the following years and will be overlooked at time of sampling, resulting in a severe sampling bias.

Recently, Esselstyn et al. provided a study assessing the reliability of the GMYC model in simulation and on a real data set of DNA sequences from Philippine bats [131]. They found a reciprocal relation between accuracy of species prediction and effective population size and/or speciation rate. Only in data sets with low haploid effective population sizes and with low speciation rates, the GMYC model predicted species limits correctly. Whereas the single threshold model switched between over- and underestimation of the number of species depending in the settings of the simulations, the multiple threshold model tended to overestimate the numbers of species [131]. As possible reasons for GMYC to fail, Esselstyn et al. mentioned the unrealistic assumptions underlying the Yule and the coalescent models (constant speciation rates without extinction in the Yule and panmictic populations of constant size in the coalescent model) [131]. Thus, not only information about biogeographical distribution or morphology, but also about effective population sizes and speciation rates are required to properly interprete the results of a GMYC analysis.

### Conclusions: DNA Barcoding in Protists

Whether the GMYC model will become more reliable in larger cryptophyte or other protistan data sets remains to be tested in future. Effective population sizes especially of planktonic protistan species tend to be large and speciation rates may be high due to short generation times at least in some lineages. Thus, considering the results of the simulation study by Esselstyn et al., the opposite effect may be encountered [131]. In addition, the vulnerability of GMYC against undersampling will likely affect data sets containing a high number of rare species, that can not be sampled densely [119]. It is also obvious, that the phylogeny-based GMYC model will fail to predict species limits properly in naturally non-monophyletic species [9]. A lack of possibilities for plausibility controls in protistan lineages (distinctive morphological characters, knowledge about biogeographical distribution, effective population sizes and speciation rates) rather speak against using the GMYC model for an automatic species delimitation. Rigorous testing of the GMYC model will be possible only in morphologically well identifiable protistan groups or in groups for which biological species limits can be tested in mating experiments. Possibly searching for a or several transition thresholds is as arbitrary as searching for a barcode gap, but perhaps an adjustment of the underlying models of speciation and coalescence will improve the GMYC model. Also the effects of constraining non-clock like and potentially non-dichotomous trees to evolve according to a molecular clock and in a strictly bifurcating pattern are not clear at all.

DNA barcoding doesn’t seem to be suited to solve problems in systematics or to define species based soley on short and highly variable DNA tags. The latter is related to the problems inherent in automatic species delimitation discussed above. Nevertheless, species identification methods can be expected to work properly in known species. Even a simple search method probably will work accurately, provided the reference database is well sampled and filled with sequences of high quality. However, different methods may be differently vulnerable to sequencing or alignment errors, which apparently also depends in the chosen barcode marker. Since next generation methods became notorious for higher error rates than Sanger sequencing [132], the influence of such errors on species identification also have to be examined in greater detail and assessed for each potential DNA barcode separately.

The establishment of an accurately working species identification system, however, throws protistan researchers back to time-consuming and tedious work not compatible with current speed at which new sequences are generated. The first step towards an accurate barcoding system in a protistan group requires integrative taxonomy by examining morphology and molecular phylogeny covering species diversity as best as possible. This is best achieved in examining clonal cultures, but establishment and maintenance of strains will not be possible in all groups. An alternative could be the isolation of single cells from field samples, their morphological examination and subsequent single-cell PCR as proposed by Auinger et al. or a mixture of examining clonal cultures and field material [38] [133]. Since also the usually longer and more conserved phylogenetic markers may be subject to the problems encountered in barcode markers (incomplete lineage sorting, compositional biases, pseudogenes, unequal evolutionary rates, intragenomic variation, gene duplications), the best option probably will be to sequence at least two, better more, unlinked genes to rule out incongruences and to use them thereafter for supermatrix trees. A species-level taxonomic revision, necessarily has to include DNA regions sufficiently variable to facilitate an identification of species. If unique molecular signatures derived from one or several genes have been included as diagnostic characters into the species descriptions, these could of course also be used as DNA barcodes in character-based identification methods. This will work only, if species are monophyletic, since non-monophyletic species require complex workarounds (e.g. different sets of molecular signatures for one species). Only in biological species, however, natural paraphylies may occur due to hybridizations or due to isolated island speciation. A taxonomic revision of protists usually confronts a researcher with the problem of how to reproducibly delimit species in groups that can not be subjected to crossing experiments or that propagate only asexually, and at the same time lack resolution at morphological level, but are genetically too diverse to be merged to one species [134].

The next step to establish a DNA barcoding system could be testing of additional candidate barcode markers, if no suitable DNA region was included in the taxonomic revision. Coleman’s species concept of compensatory base pair changes is based on the secondary structure of internal transcribed spacer 2. For this part of the ribosomal operon, intragenomic variation has been reported that may complicate its use as a barcode marker [135]–[137]. Consequently, an accurate identification by ITS2 would require sequencing of all variants present in the protists’ genomes. A supermatrix tree from a taxonomic revision facilitates identification of problems in barcode markers or in identification methods by comparing the two trees. During this phase of establishing a barcode system, failures in species identification as demonstrated in this study may be most likely, when using the blast algorithms to confirm the identity of sequences at genus or higher classification levels.

The last step requires setting up of a database and implementation of a reliable search algorithm. It cannot be taken for granted that character-based methods using even shorter tags e.g. the V4 region of the SSU rRNA gene will perform better than COI-5P in a database crowded with sequences from all kinds of organisms [7] [138]. Identification failures could be reduced by restricting searches to the group of interest. This, however, will not work in biodiversity surveys based on sequencing environmental DNA with standard cross-eukaryote primers. This requires PCR amplification with group-specific primers as has been done e.g. for the Foraminifera or the haptophytes [7] [138]. If DNA barcoding is supposed to work reliably across eukaryote kingdoms, a set of molecular markers resolving at different levels could be the best option. Isolating of single cells from field samples, e.g. by fluorescence-activated cell sorting and multi-plex PCR allows for a safe assignment of all markers to the same individual cell. It seems, though, that no fast and easy bypasses for systematics and DNA barcoding can be currently recommended for protistan researchers. The automatic species delimitation methods tested in this study proved to be highly dependent in taxon sampling and prone to artifacts.

## Materials and Methods

### Algal Cultures

Clonal cultures of cryptophytes were grown under light dark cycles of 14 10 h at either 16°C or at 23°C list of strains: File S1). As culture media either a modified WARIS-H medium (freshwater strains) or modified versions of the artificial seawater medium ASP-2 (marine and brackish water strains) have been used [139]–[142]. Instead of the usual trace metals recipes, the richer trace metals solution of the L1 medium has been added to both freshwater and marine media (WARIS-H: 100 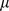
L/L; ASP-2∶1 mL/L) [143]. The different ASP-2 versions contained either no additives or soil extract (1 ml/L) or 31.7 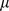
M Na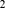-glycerophosphate.

### DNA Isolation, PCR and Sequencing

For DNA isolation, resuspended cell pellets obtained from centrifugation (5 min. at 18,000×g) of up to 8 mL of culture have been pipetted onto Whatman FTA® Mini cards (VWR, Darmstadt, Germany). Prior to PCR, small disks of 2 mm diameter were stanced out and washed in 0.2 mL PCR tubes according to the manufacturers protocol. To the DNA – still attached to the paper carrier – the PCR master mix was added. Nuclear partial LSU rDNA sequences (5′ terminus of the nuclear LSU rDNA, comprising domains A to C and parts of D) have been PCR-amplified with a nucleus-specific primer combination and sequenced completely double-stranded using previously established procedures (for accession numbers, see File S1) [56].

At time of primer construction, only three of the four cryptophyte COI-5P or *cox*1 sequences have been available in the joint EMBL/GenBank/DDBJ databases (complete mitochondrial genomes of *Hemiselmis andersenii* strain CCMP644, acc. no. EU651892 and of a not specified *Rhodomonas salina* strain, acc. no. AF288090, and a partial *cox*1 gene of *Cryptomonas ovata* strain NIES-274, acc. no. AB009419). These sequences were used to design PCR and sequencing primers for cryptophyte COI-5P sequences >500 nt (File S2). Due to the high variability of the gene, possibilities to construct a more stable forward primer have been restricted, thus annealing temperature had to be reduced to 50 C. The resulting COI-5P sequence of *Cryptomonas curvata* strain CCAC 0080 has been submitted under the accession no. HE855366 to EBI-EMBL.

### Data Analyses

Blast searches have been performed with unpruned sequences. The newly obtained COI-5P sequence of *Cryptomonas curvata* strain CCAC 0080 and several 5′-partial nuclear LSU rDNA sequences representing new lineages at different classification levels in different cryptophyte clades have been used as query sequences to test the performance of the different nucleotide versus nucleotide search algorithms offered at NCBI [78]. The partial LSU rDNA sequences represented either a species of the *Rhodomonas* clade not yet available in GenBank (1 sequence), new lineages in the *Chroomonas clade* (2 sequences) or a new sub-lineage within the species *C. curvata* (1 sequence). According to Kim et al. the codon usage in the mitochondrial genome of the cryptophyte *Hemiselmis andersenii* strain CCMP644 corresponded to the standard code table, thus this setting was used for a blastx search with the *C. curvata* CCAC 0080 COI-5P sequence as a query [80].

For phylogenetic analyses and frequency distributions, two different alignments have been assembled from 5′-partial LSU rDNA sequences. The *Cryptomonas* data set comprised 64 OTUs, including 8 new sequences, the *Chroomonas* data set 45 sequences, including 11 new sequences (File S1). Both alignments contained no outgroup taxa and were automatically pre-aligned with MUSCLE [144]. Alignment errors have been corrected by eye using the multiple sequence alignment editor SeaView 4.3.3 [145]. Non-alignable regions have been excluded for phylogenetic analyses, distance computations and saturation tests. The final *Cryptomonas* data set consisted of 975 and the *Chroomonas* data set of 920 positions. Phylogenetic analyses have been performed using the threaded version of RAxML 7.2.6 (maximum likelihood) and the MPI version of MrBayes 3.1.2 (Bayesian analyses) [146]–[147]. For maximum likelihood analyses, GTR+I+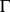 has been used as an evolutionary model including 1000 bootstrap replicates for each data set. MrBayes was set to 2 runs with four chains each, 4 million generations and GTR+I+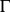. The burn-in phase for each data set has been determined and removed using the “sump” command.

To compare genetic distances and saturation among COI-5P sequences, the *C. curvata* CCAC 0080 COI-5P sequence, all cryptophyte sequences and their neighboring sequences found one position up and down in the discontiguous megablast ranking have been aligned (12 sequences, see results and Table 3) and subjected to comparative distance analysis with the K2P and GTR+I+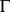 models. All three alignments, the small COI-5P alignment as well as the two 5′-partial LSU rDNA data sets have been subjected to tests for substitution saturation [66]–[67]. Substitution saturation of the data sets has been examined using the test according to Xia et al. in DAMBE 5.2.57 under exclusion of gaps [67].

Genetic distances have been computed with Paup 4.0b10 [148]. For a comparison of the effects of different distance measures on frequency distributions, four different distance measures have been inferred from the *Chroomonas* data set. For computation of the two most common distance measures in DNA barcoding, uncorrected p- and Kimura-2-parameter distances, the algorithms implemented in Paup under the distance criterion have been used. A run of jModeltest 0.1.1 yielded TIM2+I+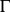 as the best trade-off between complexity and appropriate approximation of molecular evolutionary processes [68]. For distances under the latter and under the most complex evolutionary model, GTR+I+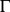, the respective maximum likelihood parameters have been estimated given a Jukes-Cantor neighbor-joining tree and thereafter used for distance computation. For the *Cryptomonas* data set intra- and interspecific GTR+I+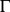 distances have been coded separately to yield frequency distributions for each. The distances inferred from both data sets were exported from Paup as column formatted text files and were imported into OpenOffice.org Calc 3.2.1 for further processing [149]. The genetic distances were sorted into distance classes to generate frequency distributions for the *Cryptomonas* and *Chroomonas* data sets. This procedure did not include computation of mean values or standard deviations to avoid biased frequency distributions.

For GMYC at first Bayesian analyses with BEAST 1.7.2 have been performed to obtain an uncalibrated tree under the assumption of a molecular clock [150]. To account for all possible clock models, the random local clock setting has been chosen and clock rates were estimated [121]. Tree prior was set to Yule process [151]–[152]. Two Markov chain Monte Carlo runs with 40 million generations and sampling of every 1000th generation have been performed to increase effective sample sizes (ESS) for each run beyond 200. Burn-in was determined with Tracer 1.5.0 (400,000 and 17,000,000 generations, respectively) [153]. After excising the trees drawn during burn-in process, the samples of two runs have been merged. The representative tree selected by the treeannotator modul of the BEAST software suite was imported into the statistics software R 2.15.1 [154]. GMYC analysis required the R package SPLITS obtainable from the R-Forge website [63] [73]. The results of the analyses (ultrametric tree plot and semi-logarithmic lineage-through-time plot with identified thresholds) have been saved in portable document format (PDF) and thereafter processed with Inkscape 0.47 [155].

## Supporting Information

File S1 **Nuclear 5′-partial LSU rDNA: list of taxa with accession numbers to database entries.**
(PDF)

File S2 **PCR and sequencing primers for the COI-5P region in cryptophytes.**
(PDF)
