# Supplementary material for: Pitfalls of Establishing DNA Barcoding Systems in Protists: The Cryptophyceae as a Test Case
Source: PLoS One. 2012 Aug 24;7(8):e43652. doi: 10.1371/journal.pone.0043652 (PMC3436593; doi:10.1371/journal.pone.0043652)
Supplement: File S1 — Nuclear 5′-partial LSU rDNA: list of taxa with accession numbers to database entries. (PDF) [file pone.0043652.s001.pdf]

Supporting File S1. Nuclear 5'-partial LSU rDNA: list of taxa with accession numbers to database entries.

Strain designation	Species name	Origin	Isolator (year of isolation)	acc. no. of partial nuclear LSU rDNA
***Cryptomonas* strains**				
CCAC 0006 (M0420)	*Cryptomonas curvata*	UK, Cornwall, Trenant	Melkonian M (1978)	AJ566147
CCAC 0024 (M0923)	*C. pyrenoidifera*	Germany, Rhein-Sieg-Kreis, Wahnbach reservoir (Wahnbachtalsperre)	Powalowski U (1992)	AJ566145
CCAC 0031	*C. obovoidea*	Germany, Bergisches Land, drying up lake close to Schommelsnaaf	Hoef-Emden K (1994)	AJ566166
CCAC 0032	*C. pyrenoidifera*	Germany, Cologne, Wahner Heide, shady puddle	Hoef-Emden K (1994)	AJ566143
CCAC 0056	C. p*aramaecium* (heterotrophic lineage 1)	Canada, Cape Breton Highlands National Park, Lake Warren, beach	Hoef-Emden K (1994)	AJ566158
CCAC 0058 (M0789)	*C. erosa*	Germany, Spessart, Biebergemünd-Bieber, pond close to Lochmühle	Schilke A (1990)	AM396384
CCAC 0080	*C. curvata*	Germany, Münster, moat of Rüschhaus	Hoef-Emden K (1995)	AJ566148
CCAC 0086	*C. marssonii*	Germany, Münster, moat of castle	Hoef-Emden K (1995)	AJ566155
CCAC 0103	*C. marssonii*	Germany, Cologne, lake district Fühlinger See, regatta lake	Hoef-Emden K (1995)	AJ715444
CCAC 0104	*C. marssonii*	Germany, Helgoland, freshwater pond	Hoef-Emden K (1996)	AJ566156
CCAC 0107 (M0850)	*C. lundii*	Germany, Cologne, University of Cologne, pond in the old nursery	Reize IB (1991)	AJ566161
CCAC 0108	*C. gyropyrenoidosa*	Germany, Dörpetal reservoir close to Remscheid	Hoef-Emden K (1993)	AJ566154
CCAC 0113	*C. borealis*	Germany, Cologne, University of Cologne, pond in the old nursery	Hoef-Emden K (1993)	AJ566160
CCAP 977/1	*C. paramaecium* (heterotrophic lineage 1)	UK, Cambridge, garden pond	Pringsheim EG (1940)	AJ715445
CCAP 977/2a	*C. paramaecium* (heterotrophic lineage 1)	unknown	Pringsheim EG (pre 1940)	AJ715446
CCAP 979/25	*C. curvata*	unknown	Pringsheim EG (unknown)	AJ715443
CCAP 979/46	*C. obovoidea*	unknown	Pringsheim EG (unknown)	AJ566167
CCAP 979/61	*C. pyrenoidifera*	Czechia, Hirschberg (Doksy?), Musikantenteich (pond)	Pringsheim EG (unknown)	AJ566142
CCAP 979/62	*C. curvata*	UK, Cumbria, Priest Pot	Jaworski GHM (1979)	AJ566150
CCAP 979/67	*C. erosa*	Finland, Vaha-Valkjari, acidic lake	Jaworski GHM (1987)	AJ566162
CCMP152	*C. pyrenoidifera*	Australia, Victoria, pond in a field	Beech P (1982)	AJ566140
M0739	*C. commutata*	Austria, mount Überling, bog	Schilke A (1989)	AJ566165
M0740	*C. erosa*	Austria, close to mount Überling, lake Seetaler See	Schilke A (1989)	AM396382
M0790	*C. erosa*	Germany, Spessart, Biebergemünd-Bieber, pond near Lochmühle	Schilke A (1990)	AM396385
M0847	*C. ovata*	Germany, Spessart, Biebergemünd-Bieber, pond near Lochmühle	Reize IB1990	AJ566153
M1077	*C. pyrenoidifera*	Germany, Cologne, University of Cologne, pot in the old nursery	Hoef-Emden K (1993)	AJ566144
M1092	*C. tetrapyrenoidosa*	Germany, lake in a beech forest next to Remscheid	Hoef-Emden K (1993)	AJ566146

M1097	*C. ovata*	Germany, Spessart, Wiesen, lake Wiesbüttsee	Hoef-Emden K (1994)	AJ566151
M1171	*C. ovata*	Austria, Burgenland, Bad Tatzmannsdorf, concrete basin	Kusel-Fetzmann E (1994)	AJ566152
M1484	*C. curvata*	Germany, Brandenburg, lake Schlachtensee, sample from deep water	Gervais F (1990)	AJ566149
M1488	*C. curvata*	unknown	Marin B (1997)	**HE820904**
M1489	*C. curvata*	unknown	Marin B (1997)	**HE820905**
M1490	*C. curvata*	unknown	Marin B (1997)	**HE820906**
M1634	heterotrophic lineage 2	Denmark, Sealand	Hoef-Emden K (1998)	AJ715457
M1639	*C. tetrapyrenoidosa*	Denmark, Jutland, Blidsø	Hoef-Emden K (1998)	AJ715454
M1799	*C. curvata*	Germany, Cologne, lake district Fühlinger See, lake no. 4	Hoef-Emden K (2000)	**HE820907**
M1800	*C. tetrapyrenoidosa*	Germany, Cologne, lake district Fühlinger See, lake no. 4	Hoef-Emden K (2000)	**HE820908**
M1975	*C. commutata*	Germany, Münster, moat of castle Hülshoff	Melkonian M (2002)	AM396380
M1980	*C. commutata*	Germany, Münster, moat of castle Hülshoff	Melkonian M (2002)	AM396381
M2061	*C. erosa*	Germany, Eifel, Dahlem, squeezing sample of *Sphagnum*	Melkonian M (2002)	AM396386
M2088	*C. loricata*	Germany, Harz, Brunnenbachweg close to Braunlage, puddle	Melkonian M (2002)	AJ715456
M2089	single-strain lineage	Germany, Harz, Brunnenbachweg close to Braunlage	Melkonian M (2002)	AM396395
M2180	*C. paramaecium* (heterotrophic lineage 1)	Germany, Cologne, Wahner Heide	Melkonian M (2002)	AJ715451
M2193	PyrX lineage	Germany, Eifel, Genfbachtal close to Engelgau	Melkonian M (2003)	**HE820909**
M2195	PyrX lineage	Germany, Eifel, Genfbachtal close to Engelgau	Melkonian M (2003)	AM396393
M2201	*C. phaseolus*	Germany, Cologne, Wahner Heide, lake Entenkaule	Melkonian M (2003)	AM396391
M2287	*C. phaseolus*	Germany, Cologne, Wahner Heide, river Agger	Melkonian M (2003)	AM396392
M2289	*C. loricata*	Germany, Cologne, Wahner Heide, river Agger	Melkonian M (2003)	AM396387
M2290	PyrX lineage	Germany, Cologne, Wahner Heide, lake Entenkaule	Melkonian M (2003)	AM396394
M2452	*C. paramaecium* (heterotrophic lineage 1)	Germany, Eifel, Kall, artificial pond close to supermarket	Hoef-Emden K (2003)	AJ715452
M2504	single-strain lineage	Germany, Cologne, Wahner Heide	Melkonian M (2003)	AM396396
M2587	*C. lundii*	Sweden, lake Möckelnsee	Melkonian M (2003)	**HE820910**
M2807	single-strain lineage	Germany, Spessart, puddle in a bog close to source of river Bieber	Klingberg M (1998?)	AM396397
M2808	*C. borealis*	Germany, Thuringia	Klingberg M (unknown)	**HE820911**
M2811	*C. obovoidea*	Germany, Spessart, fire water pond close to river Haßbach	Klingberg M (1998?)	AM396560
M2812	*C. loricata*	Germany, Spessart, puddle at the margin of Wiesbüttmoor (bog)	Klingberg M (1998?)	AM396388
M2814	*C. loricata*	Germany, Spessart, puddle in a bog close to source of river Bieber	Klingberg M (1998?)	AM396389
NIES-279	*C. tetrapyrenoidosa*	Japan, Hiroshima, Higashihiroshima	Ishimitsu M (1983)	AJ715455
SAG 2013	*C. phaseolus*	Germany, Berlin-Zehlendorf, lake Schlachtensee	Gervais F (1990)	AJ566157
SAG 977-2e	*C. paramaecium*	Germany, Bad Driburg, pond in the park	Pringsheim EG (1957)	AJ715449

	(heterotrophic lineage 1)			
SAG 977-2f	heterotrophic lineage 3	South Africa, Ottery Road, Station	Pringsheim EG (1959)	AJ715458
SAG 977-2i	*C. paramaecium* (heterotrophic lineage 1)	Switzerland	Pringsheim EG (1960)	AJ715450
SCCAP K-0063	*C. borealis*	Denmark, Jutland, Lildstrand	Christensen T (1966)	AJ566159
UTEX 2194	*C. obovoidea*	USA, Pellston, Douglas Lake	Fuller DR (1978)	AJ566168
**Strains of the *Chroomonas* clade**				
ACOI 1366	*“Chroomonas coerulea”*	Portugal, Coimbra, Convento de Sta Clara-a-Velha, tank	Carvalho G? (2001)	**HE820912**
CCAP 978/03	“*Chroomonas* sp.”	UK, Wales	Pringsheim EG (unknown)	AM901311
CCAP 978/08	“*Chroomonas placoidea*”	UK, Yorkshire	Butcher RW (1959)	AM901313
CCAP 984/02	*Hemiselmis rufescens*	English Channel	Parke M (1949)	AM901312
CCMP268	“*Chroomonas pauciplastida*”	USA, Massachusetts, Nantucket Sound, Woods Hole	Provasoli L (1956)	AM901314
CCMP269	“*Chroomonas mesostigmatica*”	USA, Massachusetts, Nantucket Sound, Woods Hole, eel pond	Guillard R (unknown)	AM901315
CCMP270	“*Chroomonas* sp.”	USA, Massachusetts, Nantucket Sound, Woods Hole, eel pond	Keller M (1982)	AM901316
CCMP439	*Hemiselmis andersenii*	Nord Atlantik, Golf von Mexiko, Cape San Blas	unknown (unknown)	AM901317
CCMP440	*Hemiselmis rufescens*	USA, Maine, West Boothbay Harbor, Bigelow Laboratory Dock	Keller M (1987)	AM901318
CCMP441	*Hemiselmis andersenii*	North Atlantic, Gulf of Mexico, gulf stream	Provasoli L (1981)	AM901319
CCMP443	*Hemiselmis tepida*	USA, Texas, Galveston Channel	Provasoli L (unknown)	AM901320
CCMP644	*Hemiselmis andersenii*	North Atlantic, Gulf of Mexico, gulf stream	Provasoli L (unknown)	AM901321
CCMP706	*Hemiselmis pacifica*	USA, Washington, San Juan Island, Friday Harbor	unknown (unknown)	AM901322
CCMP1168	“*Chroomonas* cf. *mesostigmatica*”	unknown	unknown (unknown)	AM901323
CCMP1180	*Hemiselmis andersenii*	North Atlantic, Gulf of Mexico	Provasoli L (1981)	AM901324
CCMP1181	*Hemiselmis cryptochromatica*	USA, Maine, West Boothbay Harbor, Bigelow Laboratory Dock	Selvin R (1986)	AM901325
CCMP1221	“*Chroomonas* sp.”	unknown	unknown (unknown)	AM901326
M0851	“*Chroomonas* sp.”	Germany, Cologne, University of Cologne, pond in the old nursery	Reize IB1991	AM901327
M1074	“*Komma caudata*”	Germany, Cologne, University of Cologne, concrete basin next to the University's main library	Hoef-Emden K (1993)	AM901329
M1312	“*Chroomonas* sp.”	Germany, Münster, former sewage field, squeezing sample	Hoef-Emden K (1995)	AM901330
M1318	“*Chroomonas* sp.”	France, Bretagne, Ile de Batz	Marin B (unknown)	AM901331
M1481	“*Chroomonas* sp.”	Germany, Spessart, Biebergemünd, river Bieber	Leukart P (1994)	AM901332
M1624	“*Chroomonas* sp.”	Denmark, Sealand, Virum, Kollelev Mose	Hoef-Emden K (1998)	AM901333
M1627	“*Chroomonas* sp.”	Denmark, Sealand, Bellevue Strandbad	Hoef-Emden K (1998)	AM901334

M1635	*Hemiselmis virescens*	Sweden, Kristineborg	Hoef-Emden K (1998)	AM901335
M1703	"*Chroomonas* sp."	Denmark, Jutland, Hjerting near Esbjerg	Hoef-Emden K (1998)	AM901336
M1953	"*Chroomonas* sp."	Germany, Münster, moat of castle Hülshoff	Feja N (2002)	AM901337
M2067	"*Chroomonas* sp."	Germany, Eifel, Dahlem, bog Heidemoor	Melkonian M (2002)	AM901338
M2291	"*Chroomonas* sp."	Germany, Cologne, Wahner Heide, river Agger	Melkonian M (2003)	AM901339
M3416	"*Chroomonas* sp."	Germany, Cologne, Wahner Heide, puddle	Hoef-Emden K (2008)	**HE820913**
NIES-706	"*Chroomonas nordstedtii*"	Japan, Nagano, Sugadaira Mire	Inouye I (1976)	**HE820914**
NIES-707	"*Chroomonas nordstedtii*"	Japan, Chiba, Funada-ike	Erata M (1985)	**HE820915**
NIES-708	"*Chroomonas nordstedtii*"	Japan, Sapporo, Hokkaido University	Erata M (1987)	**HE820916**
NIES-711	"*Chroomonas nordstedtii*"	Japan, Ibaraki, Mitsukaido	Suda S (1987)	**HE820917**
NIES-712	"*Chroomonas caudata*"	Japan, Chiba, Funada-ike	Erata M (1985)	**HE820918**
NIES-713	*"Chroomonas coerulea"*	Japan, Nagano, Sugadaira Mire	Erata M (1985)	**HE820919**
NIES-714	*"Chroomonas coerulea"*	Japan, Nagano, Sugadaira Mire	Erata M (1985)	**HE820920**
NIES-1004	*"Chroomonas coerulea"*	Japan, Hokkaido, Tomakomai, river Bibi	Mayumi M (1999)	**HE820921**
NIES-1370	"*Chroomonas mesostigmatica*"	Japan, Wakayama, Isonoura beach	Atsushi K (2004)	**HE820922**
SAG 980-1	"*Chroomonas* sp."	UK, Wales	Pringsheim EG (1945)	AM396398
UTEX 2000	"*Chroomonas* sp." (*Hemiselmis* sp.)	USA, Virginia, York River, Gloucester Point	Ott FD (1974)	AM901340
UTEX 2002	*Hemiselmis virescens*	USA, Virginia, York River, Gloucester Point	Ott FD (1974)	AM901341
UTEX 2779	"*Chroomonas pochmanni*"	USA, Colorado, Larimar, Wellington Reservoir #4	Kugrens P (unknown)	AM901342
UTEX 2780	*"Chroomonas coerulea"*	USA, Colorado, Glenwood Springs, Sheldon Lake	Kugrens P (unknown)	AM901343
***Rhodomonas* strain**				
CCAP 978/13	*"Chroomonas salina"*	UK, Essex, River Crouch Estuary, Fambridge marshes	Butcher RW (1956)	**HE820923**

Accession numbers of new sequences in bold face. Species names of *Cryptomonas* and *Hemiselmis* strains according to previous revisions (Hoef-Emden and Melkonian 2003; Hoef-Emden 2007; Lane and Archibald 2008). Species names in quotation marks according to culture collection entries (have not been subject to a revision). Strain UTEX 2000 is a *Hemiselmis* species. ACOI, Algoteca de Coimbra (Coimbra Collection of Algae; Coimbra, Portugal); CCAC, Culture Collection of Algae at the University of Cologne (Cologne, Germany); CCAP, Culture Collection of Algae and Protozoa (Oban, UK); CCMP, The Provasoli-Guillard National Center for Culture of Marine Phytoplankton (West Boothbay Harbor, Maine, USA); M, algal culture collection Melkonian at the University of Cologne (Cologne, Germany); NIES, Microbial Culture Collection at the National Institute for Environmental Studies (Tsukuba, Japan); SAG, Sammlung von Algenkulturen Göttingen (SAG Culture Collection; Göttingen, Germany); SCCAP, Scandinavian Culture Collection of Algae and Protozoa (Copenhagen, Denmark); UTEX, The Culture Collection of Algae at The University of Texas at Austin (Austin, Texas, USA)
