# Supplementary material for: Pitfalls of Establishing DNA Barcoding Systems in Protists: The Cryptophyceae as a Test Case
Source: PLoS One. 2012 Aug 24;7(8):e43652. doi: 10.1371/journal.pone.0043652 (PMC3436593; doi:10.1371/journal.pone.0043652)
Supplement: File S2 — PCR and sequencing primers for the COI-5P region in cryptophytes. (PDF) [file pone.0043652.s002.pdf]

Supporting File S2. PCR and sequencing primers for the COI-5P region in cryptophytes.

**Primer name**	**5' to 3' sequence**
Cr-cox1F	CKA CWA ATC ATA AAG ATA TTG G
Cr-cox1R ext2	CTT CTT GGC ATY CCW GCT AAA CCT
Cr-cox1R int	TAA ACT TCM GGA TGM CCA AAR AAC C

For PCR, the forward primer *Cr-cox1F* was used in a 5' biotinylated version to facilitate purification of the PCR products prior to sequencing with the Dynabead M-280 system (Invitrogen). Reverse primer *Cr-cox1R ext2* was used in primary PCR, reverse Primer *Cr-cox1R* int for semi-nested reamplification. For sequencing with the bidirectional automated Li-Cor sequencer 4200L, primers *Cr-cox1F* and *Cr-cox1*R int were used as labeled versions with 5'-attached fluorescence dyes IRDye-800 (forward) and IRDye-700 (reverse primers).
